# Antimicrobial resistance trends among dominant pathogens in six clinical departments of the Fourth Affiliated Hospital of Guangxi Medical University, 2020–2024

**DOI:** 10.3389/fpubh.2026.1836417

**Published:** 2026-06-11

**Authors:** Wentao Tang, Jieyu Yin, Zhougui Ling

**Affiliations:** Department of Respiratory and Critical Care Medicine, The Fourth Affiliated Hospital of Guangxi Medical University/Liuzhou Workers’ Hospital, Liuzhou, Guangxi Zhuang Autonomous Region, China

**Keywords:** antibiotics, antimicrobial resistance rate, dominant pathogen, nosocomial infection, trends

## Abstract

**Objective:**

A review of antibiotic resistance and pathogen detection in six key clinical departments of our hospital was conducted between 2020 and 2024. Future hospital-acquired infection prevention, control strategies, and targeted medicine administration in relevant departments can be based on this study.

**Methods:**

We performed a retrospective analysis of bacterial isolates collected from six clinical departments in our hospital over the past 5 years, focusing on the number, distribution, and changing trends in antibiotic resistance rates among common infections.

**Results:**

22,201 pathogenic bacterial strains were found in six departments over 5 years. *Acinetobacter baumannii* was the most common pathogen in the Intensive Care Unit. *Pseudomonas aeruginosa* in Pulmonary and Critical Care Medicine; *Escherichia coli* in Hematology; *Haemophilus influenzae* in Pediatrics; *Escherichia coli* in Orthopedics; *Klebsiella pneumoniae* in Neurosurgery. While *Haemophilus influenzae* showed increasing trends, the proportion of *Pseudomonas aeruginosa* among annually detected pathogens showed downward annual fluctuations (*p* < 0.05 for both); no significant linear variations were seen in prominent pathogens across other departments. The multidrug resistance rates of *Acinetobacter baumannii* remained consistently high. *Klebsiella pneumoniae* showed aberrant oscillations in amikacin resistance (*p* < 0.001) and significantly elevated resistance to cefotaxime and levofloxacin. *Escherichia coli* and *Pseudomonas aeruginosa* continued to exhibit low-to-moderate resistance rates to three antibiotics, with no discernible trends (*p* > 0.05). *Haemophilus influenzae* showed no resistance to ofloxacin or cefotaxime. The Intensive Care Unit had the highest resistance burden (*p* ≤ 0.002). The departments of Hematology, Neurosurgery, Orthopedics, and Respiratory and Critical Care Medicine are at an intermediate level; Pediatrics has the lowest burden of drug resistance.

**Conclusion:**

The distribution of infectious pathogens in our hospital varies significantly by department. The associated antibiotic resistance profiles reveal a considerable burden in high-risk departments, and some resistance patterns are worsening over time. These findings point to an urgent need for targeted, department-specific antimicrobial stewardship and infection control strategies.

## 1 Introduction

Antibiotics have been used extensively in clinical antimicrobial therapy since their development, successfully reducing the incidence and mortality of hospital-acquired infections. However, antimicrobial resistance (AMR) has progressively emerged as a global public health crisis. If left unchecked, AMR is projected to contribute to over 5 million deaths annually by 2050 (1, 2). In order to do this, the World Health Organization has urged that new antibiotics be developed more quickly and that drug-resistant pathogen surveillance be strengthened (3).

Since 2005, China has established two major clinical surveillance networks: the China Antimicrobial Surveillance Network (CHINET) and the China Antimicrobial Resistance Surveillance System (CARSS). Now, CHINET includes 77 hospitals across the country and provides nearly two decades of longitudinal data on antimicrobial resistance. While CARSS is an official national platform under the National Health Commission, covering more than 1,000 medical institutions across all 31 provinces in China (4, 5). Even with such considerable infrastructure in place, there are still some key holes. One notable issue is the severe underrepresentation of primary care facilities in current surveillance systems (6). Besides, infection profiles and resistance patterns vary considerably across clinical departments. These differences are largely explained by patient-level factors — including underlying comorbidities, immune function, frequency of invasive procedures, and cumulative prior antibiotic exposure (7–9). It is important to note that though national-level summaries are useful for developing policies, they only offer very little detail to direct empirical prescription at the departmental level.

To address this knowledge gap, our study analyzed pathogen detection and antimicrobial resistance patterns across six major clinical departments at the Fourth Affiliated Hospital of Guangxi Medical University from 2020 to 2024. These departments included the Intensive Care Unit (ICU), Pulmonary and Critical Care Medicine (PCCM), Hematology(Heme), Pediatrics(Peds), Neurosurgery(NS), and Orthopedics(Ortho). By analyzing relevant data, this study aims to supplement existing national surveillance data and provide a basis for antimicrobial stewardship at the institutional level.

## 2 Materials and methods

### 2.1 Information

The retrospective study was conducted on all pathogenic bacteria isolated from clinical specimens submitted between 01/01/2020 and 31/12/2024, and departments included ICU, PCCM, Heme, Peds, Ortho, and NS. We recorded the number, species distribution, and drug resistance rates of the bacterial isolates.

The study protocol was reviewed and approved by the Research Ethics Committee of Liuzhou Works’ Hospital, with the approval valid for the period of 2019–2024. And retrospective data collection for this study was carried out between 2020 and 2024, which falls entirely within the approved timeframe. All procedures followed the approved guidelines and the principles of the Declaration of Helsinki. The data were accessed on 16/09/2025.

### 2.2 Methods

#### 2.2.1 Specimen collection

All clinical specimens were collected and transported in strict accordance with the guidelines outlined in Standards for the Collection and Transport of Specimens for Clinical Microbiological Testing (10). This ensured standardized procedures prior to laboratory analysis. Clinical specimens submitted for bacterial culture included sputum, bronchoalveolar lavage fluid, blood, urine, wound secretions, cerebrospinal fluid, and sterile body fluids (pleural effusion, ascites). Specimen types varied by clinical department and patient condition. Specimen collection and transport followed institutional standard operating procedures.

To avoid bias from repeated sampling, we excluded duplicate isolates from the same patient. Duplicate isolates were defined as isolates of the same bacterial species with identical antimicrobial susceptibility profiles recovered from the same patient within a 14-day period, regardless of specimen source. Only the first isolate per patient per species per hospitalization episode was included in the analysis.

Isolates were assigned to clinical departments based on the patient’s location at the time of specimen collection. For patients transferred between departments during the same hospitalization, the isolate was attributed to the department where the specimen was collected.

#### 2.2.2 Bacterial identification and antimicrobial susceptibility testing

We identified all clinical isolates and performed antimicrobial susceptibility testing with the VITEK-2 automated system (bioMérieux, France). This system works on the broth microdilution principle to determine minimum inhibitory concentrations (MICs). For antimicrobial agents whose breakpoints were not covered by the automated system, we used the disk diffusion method or the E-test as a supplementary approach.

Susceptibility results were interpreted according to the breakpoints recommended in the current annual editions of the CLSI M100 document in effect during each year from 2020 to 2024. Our hospital’s clinical microbiology laboratory followed a standard practice throughout the study period: CLSI 2022 criteria (M100-S32). This practice is corroborated by institutional surveillance publications from the same period. Importantly, all susceptibility categories (susceptible, intermediate, and resistant) reported in this study reflect the updated M100-S32 (2022) breakpoints. The revised fluoroquinolone breakpoints introduced over the past decade—most notably the substantially lowered MIC thresholds for ciprofloxacin and levofloxacin against Enterobacterales (including *Escherichia coli* and *Klebsiella pneumoniae*) and for *Pseudomonas aeruginosa*—were uniformly applied to all isolates collected during 2020–2024, ensuring that the longitudinal comparisons reported here are based on a single, contemporary interpretive standard rather than on legacy thresholds.

For this study, we selected ceftazidime and cefotaxime to represent the cephalosporin class. Tobramycin and amikacin were chosen to represent the aminoglycoside class. Ofloxacin and levofloxacin were chosen to represent the fluoroquinolone class. Carbapenems were not included in the scope of this analysis.

To confirm the accuracy of the longitudinal resistance trends reported for the study period, raw MIC values exported from the VITEK-2 system were cross-verified against the CLSI M100-S32 (2022) breakpoints. Specifically, the categorical S/I/R interpretations originally generated by the VITEK-2 system between 2020 and 2024 were re-evaluated by manually re-mapping each raw MIC value to the corresponding S32 breakpoint table for the relevant organism–antimicrobial pair, with particular attention to fluoroquinolones (ciprofloxacin, levofloxacin, ofloxacin), for which the breakpoints have been substantially revised over the past decade. This cross-verification step was performed to confirm the clinical accuracy of the longitudinal trends reported for the study period (2020–2024). No discrepancies were identified between the system-generated categorical interpretations and the expected classifications based on the CLSI M100-S32 criteria.

### 2.3 Quality control

To ensure the reliability of antimicrobial susceptibility testing, daily quality control was performed using standard reference strains. We carried out quality control with a set of reference strains. Each strain was selected according to the bacterial group being tested. For Enterobacterales (including *Escherichia coli* and *Klebsiella pneumoniae*), we used *Escherichia coli* ATCC 25922. For non-fermenting Gram-negative bacilli (including *Pseudomonas aeruginosa* and *Acinetobacter baumannii*), *Pseudomonas aeruginosa* ATCC 27853 was used. For *Haemophilus influenzae* itself, the reference strain ATCC 49247/9007 served as the control.

### 2.4 Statistical analysis

We used WHONET software (version 5.6) to collate and analyze the antimicrobial susceptibility data. Antimicrobial resistance data were managed and analyzed using WHONET version 5.6 (World Health Organization). Resistance rates and confidence intervals were calculated using default analysis parameters. Breakpoints were configured according to CLSI 2022 criteria (M100-S32) (11) without customization.

Counts (*n*), percentages (%), and 95% confidence intervals were used to present the data.

For trend analysis, we applied the Cochran-Armitage test to assess changes from 2020 to 2024 in the distribution of major pathogens and their resistance rates across departments. When the test assumptions were not met (for example, when the expected frequency was below 5 or zero), Fisher’s exact test was used instead.

With regard to differences among departments, we first checked whether the data from each department followed a normal distribution and had equal variances. When both conditions were satisfied, a one-way analysis of variance (ANOVA) was performed, followed by *post hoc* pairwise comparisons with Bonferroni correction. If the conditions were not met, the Kruskal–Wallis H test was applied, and Dunn’s test (with Bonferroni adjustment) was used for subsequent pairwise comparisons.

All statistical analyses were run using SPSS software (version 22.0). We set a two-sided *p*-value below 0.05 as the threshold for statistical significance.

## 3 Results

### 3.1 Distribution of the top five pathogens by department

**Figure 1:**
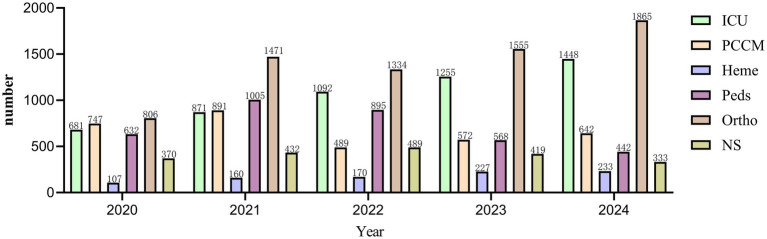
Distribution of pathogenic isolates by department over the past 5 years. ICU, the Intensive Care Unit; PCCM, Pulmonary and Critical Care Medicine; Heme, Hematology; Peds, Pediatrics; NS, Neurosurgery; Ortho, Orthopedics. A total of 3,343 pathogenic isolates were identified in 2020, followed by 4,830 in 2021, 4,469 in 2022, 4,596 in 2023, and 4,963 in 2024.

**Figure 2:**
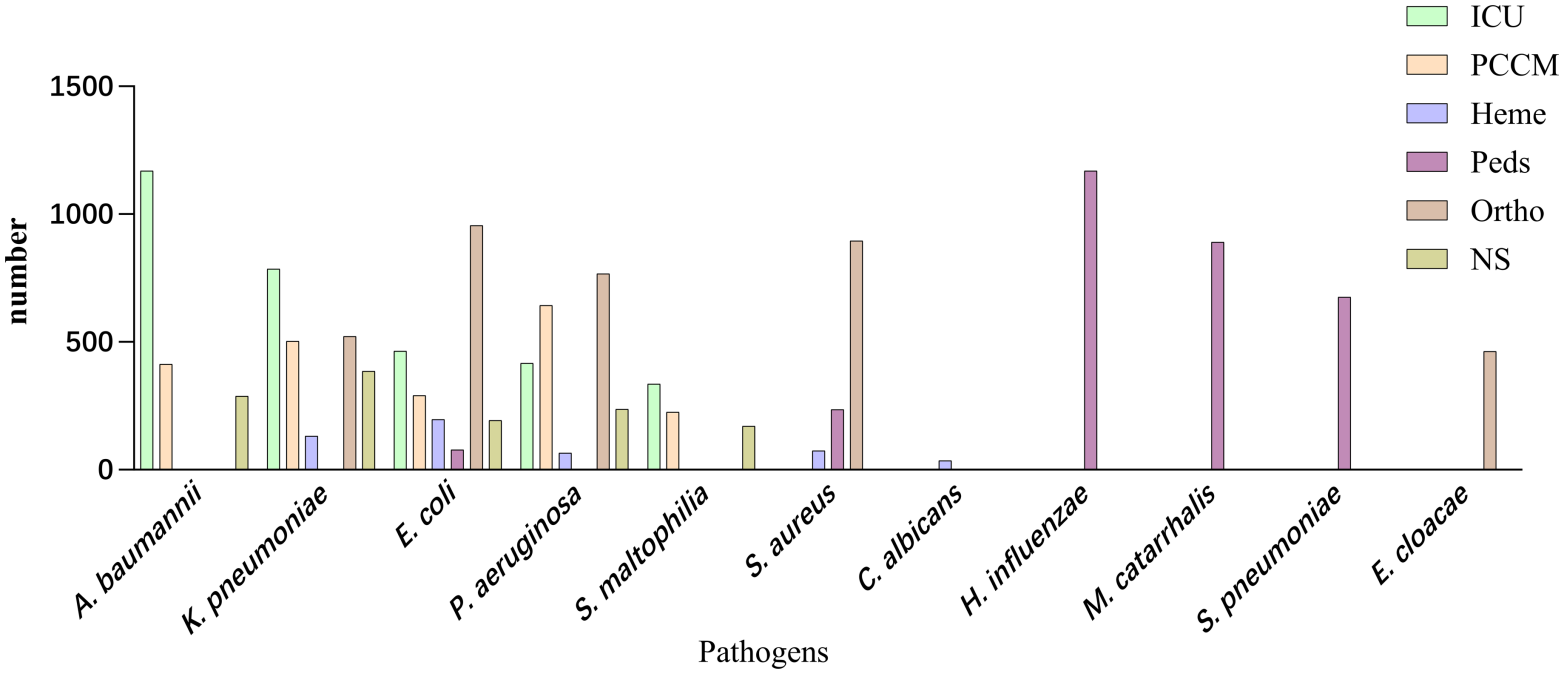
Department-specific distribution of the top five pathogenic isolates. ICU, the Intensive Care Unit; PCCM, Pulmonary and Critical Care Medicine; Heme, Hematology; Peds, Pediatrics; NS, Neurosurgery; Ortho, Orthopedics. *A. baumannii*, *Acinetobacter baumannii*; *K. pneumoniae*, *Klebsiella pneumoniae*; *E. coli*, *Escherichia coli*; *P. aeruginosa*, *Pseudomonas aeruginosa*; *S. maltophilia*, *Stenotrophomonas maltophilia*; *S. aureus*, *Staphylococcus aureus*; *C. albicans*, *Candida albicans*; *H. influenzae*, *Haemophilus influenzae*; *M. catarrhalis*, *Moraxella catarrhalis*; *S. pneumoniae*, *Streptococcus pneumoniae*; *E. cloacae*, *Enterobacter cloacae*. The top five pathogens and their respective isolate counts for each department are as follows: ICU: *Acinetobacter baumannii* (1,169), *Klebsiella pneumoniae* (785), *Escherichia coli* (464), *Pseudomonas aeruginosa* (417), *Stenotrophomonas maltophilia* (335); PCCM: *Pseudomonas aeruginosa* (643), *Klebsiella pneumoniae* (503), *Acinetobacter baumannii* (413), *Escherichia coli* (291), *Stenotrophomonas maltophilia* (225); Heme: *Escherichia coli* (197), *Klebsiella pneumoniae* (132), *Staphylococcus aureus* (74), *Pseudomonas aeruginosa* (65), *Candida albicans* (36); Peds: *Haemophilus influenzae* (1,169), *Moraxella catarrhalis* (890), *Streptococcus pneumoniae* (676), *Staphylococcus aureus* (236), *Escherichia coli* (78); Ortho: *Escherichia coli* (956), *Staphylococcus aureus* (896), *Pseudomonas aeruginosa* (767), *Klebsiella pneumoniae* (522), *Enterobacter cloacae* (463); NS: *Klebsiella pneumoniae* (385), *Acinetobacter baumannii* (288), *Pseudomonas aeruginosa* (237), *Escherichia coli* (193), *Stenotrophomonas maltophilia* (171).

A total of 22,201 pathogenic isolates were obtained from specimens collected across six departments from 2020 to 2024. The six departments showed distinct pathogen distribution patterns. In the ICU, *Acinetobacter baumannii* and *Klebsiella pneumoniae* were the most prevalent species. *Pseudomonas aeruginosa* and *Klebsiella pneumoniae* dominated in the PCCM department. The remaining four departments each had its own leading pathogen. Hematology was dominated by *Escherichia coli*; Pediatrics, by *Haemophilus influenzae*; Orthopedics, also by *Escherichia coli*; and Neurosurgery, by *Klebsiella pneumoniae*. (The complete distributions are presented in Figures 1, 2).

### 3.2 Temporal changes in predominant pathogens by department over the past 5 years

To further investigate the specific trends of the most prevalent pathogen (ranked first in proportion) in each of the six clinical departments over the five-year period, we applied the Cochran Armitage trend test to the isolate counts of the top ranked pathogen.

**Figure 3:**
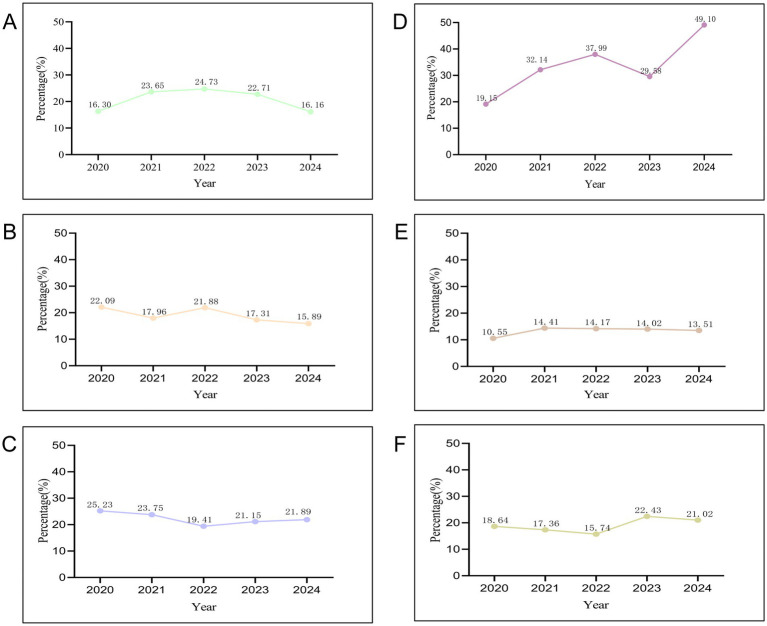
Trends in the number of predominant pathogen isolates by department (2020–2024). **(A–F)** illustrate the annual trend (2020–2024) in the proportion of the predominant pathogen relative to the total isolates within the ICU, PCCM, Heme, Peds, Ortho, and NS. The predominant pathogen for each department is as follows: ICU- *A. baumannii*, PCCM- *P. aeruginosa*, Heme- *E. coli*, Peds- *H. influenzae*, Ortho- *E. coli*, and NS- *K. pneumoniae*.

The predominant species varied by department. *A. baumannii* was the most common in the ICU; *P. aeruginosa*, in PCCM; *E. coli*, in Hematology and Orthopedics; *H. influenzae*, in Pediatrics; and *K. pneumoniae*, in Neurosurgery. Two pathogens showed statistically significant temporal trends. In PCCM, the proportion of *P. aeruginosa* fluctuated and declined from 22.09% in 2020 to 15.89% in 2024, while the proportion of *H. influenzae* in Pediatrics also fluctuated but increased from 19.15 to 49.10% over the same period (both *p* < 0.05). For the remaining pathogens, no clear linear trends were detected. These included *A. baumannii* in the ICU (*p* = 0.076), *E. coli* in Hematology (*p* = 0.471), *E. coli* in Orthopedics (*p* = 0.299), and *K. pneumoniae* in Neurosurgery (*p* = 0.114) (For detailed data, please refer to Table 1 and Figure 3).

**Table 1:** Proportion of the predominant pathogen by department over the past five years (%) (na/nb).

Dept.	Predom. Pathogen	2020	2021	2022	2023	2024	*χ*^2^ value	*p*-value
ICU	*A. baumannii*	16.30 (111/681)	23.65 (206/871)	24.73 (270/1,092)	22.71 (285/1,255)	16.16 (234/1,448)	3.150	0.076
PCCM	*P. aeruginosa*	22.09 (165/747)	17.96 (160/891)	21.88 (107/489)	17.31 (99/572)	15.89 (102/642)	7.068	0.008
Heme	*E. coli*	25.23 (27/107)	23.75 (38/160)	19.41 (33/170)	21.15 (48/227)	21.89 (51/233)	0.519	0.471
Peds	*H. influenzae*	19.15 (121/632)	32.14 (323/1,005)	37.99 (340/895)	29.58 (168/568)	49.10 (217/442)	74.840	<0.001
Ortho	*E. coli*	10.55 (85/806)	14.41 (212/1,471)	14.17 (189/1,334)	14.02 (218/1,555)	13.51 (252/1,865)	1.007	0.299
NS	*K. pneumoniae*	18.64 (69/370)	17.36 (75/432)	15.74 (77/489)	22.43 (94/419)	21.02 (70/333)	2.498	0.114

ICU, the Intensive Care Unit; PCCM, Pulmonary and Critical Care Medicine; Heme, Hematology; Peds, Pediatrics; NS, Neurosurgery; Ortho, Orthopedics. *A. baumannii*, *Acinetobacter baumannii*; *P. aeruginosa*, *Pseudomonas aeruginosa*; *E. coli*, *Escherichia coli*; *H. influenzae*, *Haemophilus influenzae*; *K. pneumoniae*, *K. pneumoniae*. In the “na/nb” notation, “na” represents the number of isolates of the corresponding predominant pathogen, and “nb” represents the total number of pathogenic isolates from that department in the given year. Since the annual total of isolates varied by department, this table records only the specific counts for the predominant pathogen each year. For the detailed annual total number of isolates (nb) per department, please refer to Figure 1.

### 3.3 Changes in resistance rates of predominant pathogens

Resistance rates of the predominant pathogens to three commonly used antibiotic classes (cephalosporins, aminoglycosides, and fluoroquinolones) were further analyzed. Two organisms stood out for their high resistance levels: *A. baumannii* and *K. pneumoniae*. Take *A. baumannii* as an example. Throughout the study period, its resistance rates to all three antibiotic classes stayed above 80%, except for a few values that fell slightly below that mark in 2020. Its resistance rates to ceftazidime and levofloxacin rose over the 5 years, even with some annual fluctuations from year to year. The Cochran-Armitage test gave a *p* value below 0.001 for both, confirming that the upward trends were statistically significant.

Now turn to *P. aeruginosa*, its resistance rates to ceftazidime, tobramycin, and levofloxacin fell between 3.0% and 40.0%. Year-to-year fluctuations were present, but no clear overall trend was detected across the 5 years (all *p* > 0.05). A similar pattern was seen for *E. coli* from Hematology, keeping low-to-moderate resistance to ceftazidime, amikacin, and levofloxacin, with no meaningful upward or downward movement (all *p* > 0.05). The same applied to *E. coli* from Orthopedics.

**Figure 4:**
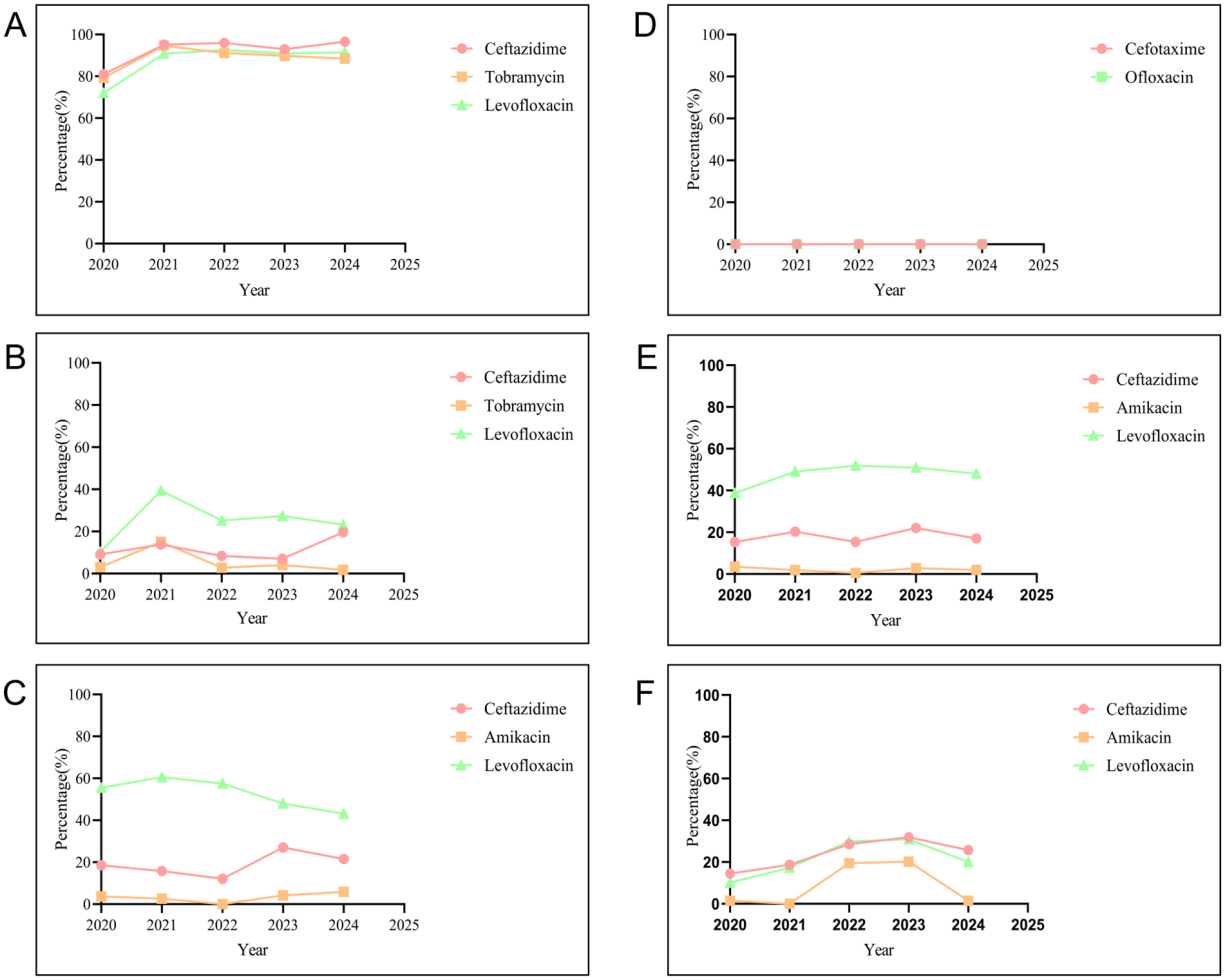
Trends in antimicrobial resistance of the predominant pathogens (2020–2024). **(A–F)** illustrate the trends in resistance rates to three antimicrobial agents for the predominant pathogens in the ICU, PCCM, Heme, Peds, Ortho, and NS departments, respectively. ICU and PCCM were tested against ceftazidime, tobramycin, and levofloxacin. *E. coli* (Heme and Ortho) and *K. pneumoniae* (NS) were tested against ceftazidime, amikacin, and levofloxacin. The sole exception was *H. influenzae* (Peds); due to the specific clinical context of pediatrics, its resistance to aminoglycosides was not assessed, and testing was performed only against cefotaxime and ofloxacin.

But Pediatrics offered a different story. Unlike the other five departments, *H. influenzae* from this unit stayed highly susceptible to both antibiotics tested (cefotaxime and ofloxacin). No resistant strains were found throughout the entire study period (For detailed data, please refer to Table 2; Supplementary Table S2; Figure 4).

**Table 2:** Trends in antimicrobial resistance of predominant pathogens to three antibiotic classes.

Dept.	Predom. Pathogen	Antimicrobial drugs	2020	2021	2022	2023	2024	*X*^2^ value	*p*-value
ICU	*A. baumannii*	CAZ	90/111 (81.08%)	196/206 (95.15%)	259/270 (95.93)	265/285 (92.98)	226/234 (96.58)	12.693	<0.001
ICU	*A. baumannii*	TOB	88/111 (79.28%)	195/206 (94.66%)	246/270 (91.11%)	256/285 (89.92)	207/234 (88.46%)	0.342	0.559
ICU	*A. baumannii*	LEV	80/111 (72.07%)	187/206 (90.78%)	250/270 (92.59%)	259/285 (90.88%)	214/234 (91.45%)	14.442	<0.001
PCCM	*P. aeruginosa*	CAZ	15/165 (9.09%)	22/160 (13.75%)	9/107 (8.41%)	7/99 (7.07%)	22/112 (19.64%)	2.500	0.114
PCCM	*P. aeruginosa*	TOB	5/165 (3.03%)	24/160 (15.00%)	3/107 (2.80%)	4/99 (4.04%)	2/112 (1.79%)	3.530	0.060
PCCM	*P. aeruginosa*	LEV	17/165 (10.30%)	63/160 (39.38%)	27/107 (25.23%)	27/99 (27.27%)	26/112 (23.21%)	2.239	0.135
Heme	*E. coli*	CAZ	5/27 (18.52%)	6/38 (15.79%)	4/33 (12.12%)	13/48 (27.08%)	11/51 (21.57%)	0.930	0.335
Heme	*E. coli*	AMK	1/27 (3.70%)	1/38 (2.63%)	0/33 (0.00%)	2/48 (4.17%)	3/51 (5.88%)	2.081	0.769
Heme	*E. coli*	LEV	15/27 (55.56%)	23/38 (60.53%)	19/33 (57.58%)	23/48 (47.92%)	22/51 (43.14%)	2.688	0.101
Peds	*H. influenzae*	CTX	0/121 (0.00%)	0/323 (0.00%)	0/340 (0.00%)	0/168 (0.00%)	0/217 (0.00%)	-	-
Peds	*H. influenzae*	OFX	0/121 (0.00%)	0/323 (0.00%)	0/340 (0.00%)	0/168 (0.00%)	0/217 (0.00%)	-	-
Ortho	*E. coli*	CAZ	13/85 (15.29%)	43/212 (20.28%)	29/189 (15.34%)	48/218 (22.02%)	43/252 (17.06%)	0.023	0.879
Ortho	*E. coli*	AMK	3/85 (3.53%)	4/212 (1.89%)	1/189 (0.53%)	6/218 (2.75%)	5/252 (1.98%)	4.175	0.356
Ortho	*E. coli*	LEV	33/85 (38.82%)	104/212 (49.06%)	98/189 (51.85%)	111/218 (50.92%)	121/252 (48.02%)	0.690	0.406
NS	*K. pneumoniae*	CAZ	10/69 (14.49%)	14/75 (18.67%)	22/77 (28.57%)	30/94 (31.91%)	18/70 (25.71%)	5.392	0.020
NS	*K. pneumoniae*	AMK	1/69 (1.45%)	0/75 (0.00%)	15/77 (19.48%)	19/94 (20.21%)	1/70 (1.43%)	41.976	<0.001
NS	*K. pneumoniae*	LEV	7/69 (10.14%)	13/75 (17.33%)	23/77 (29.87%)	29/94 (30.85%)	14/70 (20.00%)	5.059	0.024

CAZ, Ceftazidime; TOB, Tobramycin; LEV, Levofloxacin; AMK, Amikacin; CTX, Cefotaxime; OFX, Ofloxacin. *A. baumannii*, *Acinetobacter baumannii*; *P. aeruginosa*, *Pseudomonas aeruginosa*; *E. coli*, *Escherichia coli*; *H. influenzae*, *Haemophilus influenzae*; *K. pneumoniae*, *Klebsiella pneumoniae*. In this study, ceftazidime and cefotaxime were selected as representative agents for the cephalosporin class; tobramycin and amikacin for the aminoglycoside class; and ofloxacin and levofloxacin for the fluoroquinolone class. Notably, aminoglycosides are rarely used in clinical practice in the Peds; therefore, resistance monitoring for these agents was not performed for pediatric isolates. Furthermore, due to the occurrence of zero-frequency data in the amikacin resistance profiles of *E.* coli from the Heme and *K. pneumoniae* from the NS, Fisher’s exact test was applied for the respective analyses, as presented in the results above. Similarly, for the trend analysis of amikacin resistance in *E. coli* from the Ortho, four cells (40.0%) had an expected frequency of less than 5, with a minimum of 1.69, warranting the use of Fisher’s exact test. Regarding the specific antibiotic tested for each pathogen: *A. baumannii* and *P. aeruginosa* were tested against ceftazidime, tobramycin, and levofloxacin; *E. coli* and *K. pneumoniae* were tested against ceftazidime, amikacin, and levofloxacin. The sole exception was *H. influenzae*, the predominant pathogen in the Peds. Owing to the specific clinical context of this department, its resistance to aminoglycosides was not assessed. Instead, susceptibility testing for *H. influenzae* was performed only against cefotaxime and ofloxacin.

### 3.4 Inter-departmental comparison of resistance rates

Before comparing resistance rates across departments, we first checked whether the data from each department followed a normal distribution. The results showed that some departments deviated from normality (Kolmogorov–Smirnov test, *p* < 0.05), So we took the non-parametric Kruskal–Wallis H test to employ the inter-group comparisons. This analysis revealed a statistically significant difference in resistance rate distributions among the six departments (H = 553.676, df = 5, *p* < 0.001). Then, we performed pairwise comparisons between departments. For this step, we used Dunn’s *post hoc* test with Bonferroni correction.

**Figure 5:**
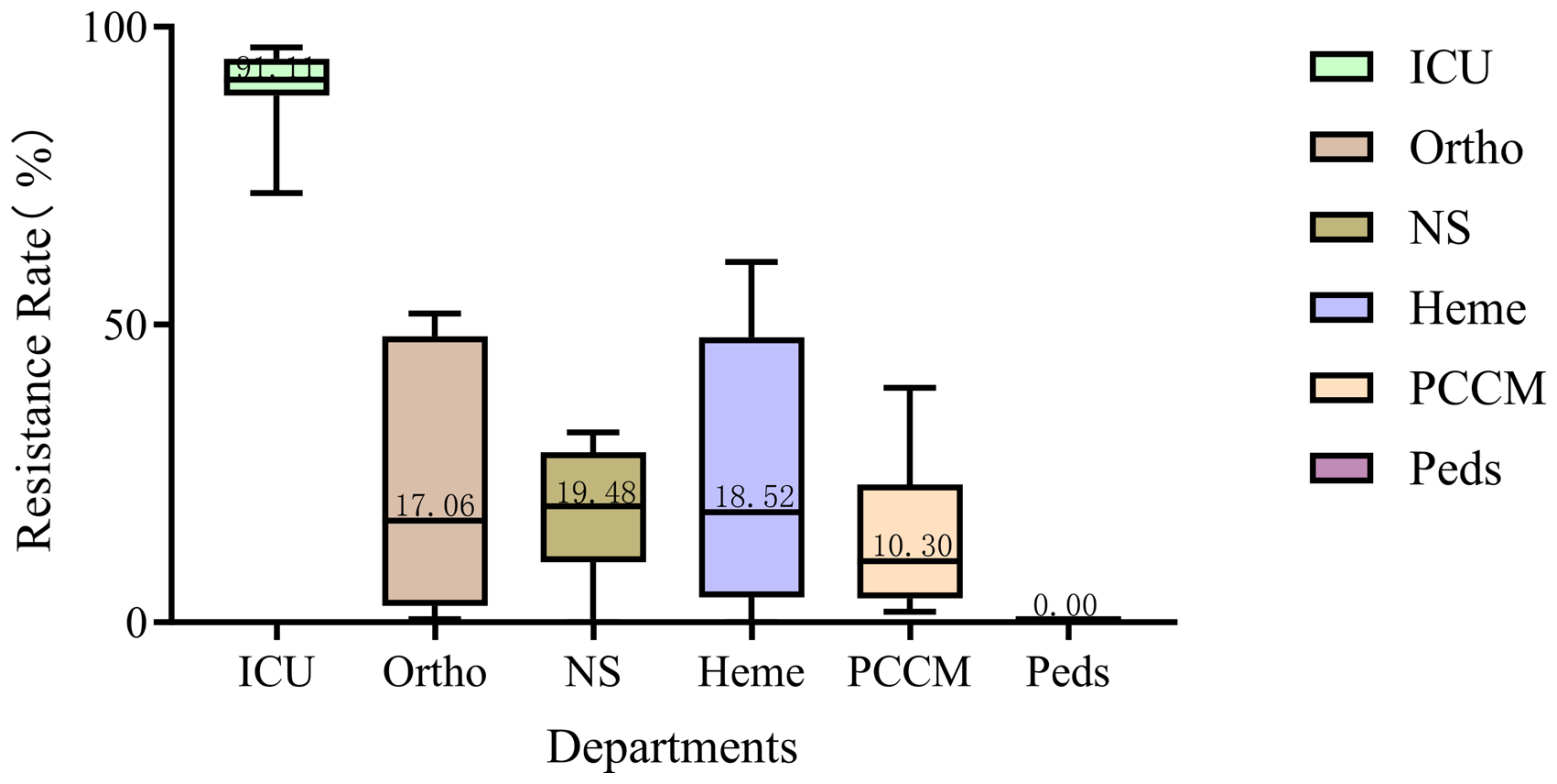
Distribution and comparison of overall bacterial resistance rates across clinical departments. ICU, the Intensive Care Unit; PCCM, Pulmonary and Critical Care Medicine; Heme, Hematology; Peds, Pediatrics; NS, Neurosurgery; Ortho, Orthopedics. *A. baumannii*, *Acinetobacter baumannii*; *P. aeruginosa*, *Pseudomonas aeruginosa*; *E. coli*, *Escherichia coli*; *H. influenzae*, *Haemophilus influenzae*; *K. pneumoniae*, *Klebsiella pneumoniae*. The box plot displays the median resistance rate (central line within the box), the interquartile range (IQR; the box itself), and the range (whiskers). Departments are arranged in descending order of their median resistance rates. The Kruskal–Wallis test indicated a statistically significant difference in resistance rates among the departments. *Post hoc* analysis using Dunn’s test with Bonferroni correction revealed that the resistance rate in the ICU was significantly higher than those in all other departments, while the rate in the Pediatrics (Peds) department was significantly lower than those in the others. No significant differences were observed among the remaining four departments.

The ICU stood out from the others, its median resistance rate was higher than that of each of the remaining five departments (all adjusted *p* ≤ 0.002). The resistance rates in Hematology, Neurosurgery, Orthopedics, and PCCM were moderate and did not differ significantly from each other (all adjusted *p* = 1.000). In contrast, the resistance rate in Pediatrics was significantly lower than that in Hematology, Neurosurgery, and Orthopedics (all adjusted *p* < 0.05). The difference between Pediatrics and PCCM was close to significance but did not quite reach it (adjusted *p* = 0.054) (For details, see Table 3; Figure 5).

**Table 3:** Pairwise comparisons of antimicrobial resistance rates among clinical departments.

Dept.	ICU	Heme	NS	Ortho	PCCM	Peds
ICU	—	0.002	<0.001	0.001	<0.001	<0.001
Heme	0.002	—	1.000	1.000	1.000	0.004
NS	<0.001	1.000	—	1.000	1.000	0.011
Ortho	0.001	1.000	1.000	—	1.000	0.007
PCCM	<0.001	1.000	1.000	1.000	—	0.054
Peds	<0.001	0.004	0.011	0.007	0.054	—

The values presented in the table are *p* values adjusted using the Bonferroni correction. An adjusted *p* value of less than 0.05 was considered statistically significant. The value at the intersection of a given row and column represents the adjusted *p* value for the pairwise comparison between the department listed in that row and the department listed in that column (e.g., the value “0.002” at the intersection of the ICU row and the Heme column indicates the adjusted *p* value for the comparison between the ICU and the Heme).

## 4 Discussion

Antimicrobial resistance in bacteria not only increases the risk of nosocomial infections but also prolongs hospital stays, escalates drug consumption, and further amplifies the economic burden on patients (12). So we carried out a retrospective study covering 2020 to 2024. The analysis focused on pathogenic isolates from six departments: ICU, PCCM, Hematology, Pediatrics, Orthopedics, and Neurosurgery. A clear pattern emerged, each department had its own dominant pathogen. *A. baumannii* led in the ICU; *P. aeruginosa* in PCCM; *E. coli* in Hematology and Orthopedics; *H. influenzae* in Pediatrics; and *K. pneumoniae* in Neurosurgery. Resistance trends also differed widely across these pathogens for cephalosporins, aminoglycosides, and fluoroquinolones. At same time, the findings tell us that infection prevention and antimicrobial stewardship should be shaped to fit each department’s specific situation. Getting this right is important for slowing the rise of resistance and for cutting down hospital-acquired infections.

### 4.1 Relationship to national AMR surveillance

This single-center, department-stratified analysis adds to what our national AMR surveillance systems already provide. CARSS and CHINET offer valuable population-level estimates of resistance across sentinel hospitals. However, their aggregated outputs may miss clinically useful patterns that only become visible at the department level (7). In this study, we analyzed 22,201 clinical isolates from six specialties over 5 years. The results show clear department-specific pathogen ecology and resistance profiles — information that is not easily seen in national reports.

Several examples illustrate the added value of more detailed, institution-level surveillance. These include: the dominance of *A. baumannii* in the ICU versus *H. influenzae* in Pediatrics; the significantly higher resistance burden in critical care settings (*p* ≤ 0.002); and the specialty-specific trends in isolation rates for *P. aeruginosa* and *H. influenzae*.

National data are useful for shaping policy and for comparing institutions. Department-level analyses, in turn, can guide local choices about empiric therapy and targeted antimicrobial stewardship. The two approaches are synergistic rather than redundant.

### 4.2 Influence of COVID-19 pandemic dynamics on observed trends

The study period from 2020 to 2024 spanned four distinct phases of China’s COVID-19 prevention and control efforts. Zhang et al. systematically defined these phases as follows: Phase 1 (the “zero-COVID” policy) was characterized by strict non-pharmaceutical interventions; Phase 2 (routine, targeted prevention and control) involved the implementation of differentiated strategies; Phase 3 (primarily targeting the Omicron variant) focused on advancing vaccination efforts and preparing medical resources; Phase 4 (the pandemic management de-escalation phase): tiered diagnosis and treatment, and coordinated allocation of medical resources (13, 14). A study from Shanghai found that lockdown measures reduced outpatient visits by 47% and hospital discharges by 55%, reflecting a major shift in medical resource allocation during the first phase (15). After the policy change in December 2022, a nationwide infection surge occurred in China. Within 1 month, the estimated cumulative number of infections reached 248 million, accounting for about 18% of the population (16).

Several department-specific trends in our data are best understood through this pandemic lens. The sharp increase in *H. influenzae* detection in Pediatrics (from 19 to 49%, *p* < 0.05) is likely explained by post-restriction “immunity debt” rather than a true rise in disease burden (17). During the Zero-COVID period, strict non-pharmaceutical interventions suppressed the transmission of *H. influenzae* and other respiratory pathogens, creating an artificially low baseline for comparison. The sharp increase in 2023–2024 corresponds to the well-documented global resurgence of pediatric respiratory infections following pandemic restriction lifting (18, 19). The proportion of *P. aeruginosa* in PCCM showed a gradual downward trend (*p* < 0.05), which may be related to enhanced environmental disinfection and contact isolation measures implemented during the “zero-tolerance” policy period (20).

Linear trend analysis revealed that the detection rate of *A. baumannii* in the ICU followed an inverted U-shaped pattern, but this was not statistically significant (*p* = 0.076). Notably, the increasing phase observed from 2020 to 2023 aligns with trends reported in other studies conducted during the same period (14). Study settings and regions varied across the reported studies, but the overall trend was consistent. Several common factors during the COVID-19 pandemic likely contributed to greater transmission and colonization of *A. baumannii*. These included ICU overload, intermittent shortages of protective equipment, increased use of broad-spectrum antimicrobials, and less frequent environmental cleaning (21, 22). We also saw a decline in detection rates at our institution starting after 2023. This downward trend may be a delayed effect of specific ICU interventions aimed at multidrug-resistant organisms. These included enhanced terminal disinfection of the environment, strict adherence to contact precautions, and optimized antimicrobial stewardship. All of these measures were implemented as the pandemic moved into a more stable phase. During major public health events, critical departments such as the ICU need early-warning and emergency-response mechanisms for resistant pathogens. Such mechanisms can help reduce nosocomial infection risks (23).

In contrast, no significant linear trends were observed in hematology (*p* = 0.471), neurosurgery (*p* = 0.114), and orthopedics (*p* = 0.299), suggesting that these specialties likely maintained relatively stable patient profiles and surgical volumes throughout the pandemic (24).

Given the profound influence of COVID-19 policies on healthcare delivery during 2020–2024, the temporal trends observed herein should be interpreted as pandemic-era patterns rather than secular AMR trajectories. Extrapolation to non-pandemic periods requires caution.

### 4.3 Antimicrobial resistance profiles of selected pathogens

Over the five-year surveillance period, we saw a consistent 0% resistance rate in *H. influenzae* isolates from the Pediatrics department for both cefotaxime and ofloxacin. This finding is quite different from the observed increase and fluctuation in detection rates. It suggests that the rise in prevalence is probably not driven by resistance factors. Other explanations seem more likely. H*. influenzae* has a limited natural ability to develop and spread resistance against third-generation cephalosporins (such as cefotaxime) and fluoroquinolones (25). Resistance in this species is mainly linked to *β*-lactamase production (e.g., TEM-1 and ROB-1). These enzymes primarily reduce the activity of ampicillin and related drugs. Third-generation cephalosporins are generally stable against them (26, 27). Fluoroquinolone resistance, on the other hand, usually comes from mutations in the gyrA and parC genes (28). In our hospital and across China, fluoroquinolone use in children is strictly limited because of potential side effects on bones and joints. These drugs are not first-line choices in pediatrics. As a result, the clinical pressure that selects for resistance is very low. The use of third-generation cephalosporins is also well controlled in this setting. Taken together, the low level of antibiotic exposure in the pediatric population makes the emergence of resistant strains much less likely. Routine susceptibility testing in clinical labs may not easily detect very rare resistance phenotypes. If the true resistance rate is extremely low (for example, below 1%), it is possible that no resistant isolates are picked up within a given sample size (29).

These findings have several practical implications. We need to maintain careful antimicrobial stewardship in pediatrics and strengthen surveillance for resistance to other classes of antibiotics. Doing so will help prevent the development of resistant organisms.

Besides, among the department-specific findings in this study, the marked fluctuation in amikacin resistance among *K. pneumoniae* isolates from Neurosurgery deserves special attention (1.45% → 0% → 19.48% → 20.21% → 1.43%; *p* < 0.001). This pattern does not fit a gradual, long-term trend in antimicrobial resistance. Instead, it points to a distinct, time-limited disruption.

There may be two key reasons for this phenomenon. One possibility is that a clonal outbreak of amikacin-resistant *K. pneumoniae* strains occurred in Neurosurgery between 2020 and 2022. Amikacin resistance in *K. pneumoniae* is often mediated by plasmid-encoded 16S rRNA methyltransferases (e.g., armA and rmtB) or by concurrent carriage of aminoglycoside-metabolizing enzymes and beta-lactamases. (30, 31). In China, the dominant carbapenem-resistant *K. pneumoniae* clone, ST11, frequently carries multiple resistance determinants, including those conferring amikacin resistance (32). Another possibility is that a temporary change in aminoglycoside prescribing practices within Neurosurgery created selective pressure that favored amikacin-resistant isolates (33). In neurosurgical patients, amikacin is sometimes used to treat healthcare-associated ventriculitis or meningitis, or as part of combination therapy for multidrug-resistant Gram-negative infections (34). A temporary increase in amikacin use could have enriched the local *K. pneumoniae* population for amikacin-resistant subclones. If that was the case, the subsequent return to baseline resistance likely reflects either a return to previous prescribing patterns or the introduction of antimicrobial stewardship measures.

Because we lacked molecular typing of the isolates and detailed antimicrobial consumption data, we could not definitively determine which of these two mechanisms was responsible. Notably, the absence of concomitant surges in resistance to other agents (e.g., carbapenems or fluoroquinolones) in Neurosurgery *K. pneumoniae* during the same period somewhat argues against a classic ST11 outbreak, which typically exhibits a multidrug-resistant phenotype. This may suggest a narrower, amikacin-specific selective pressure. In future prospective surveillance at our institution, we plan to incorporate molecular characterization of isolates that show unusual resistance patterns and to link these findings with antimicrobial consumption data. This would allow us to better understand the mechanisms behind such unusual signals in real time.

### 4.4 Limitations

First, our findings come mainly from selected departments at a single hospital over 5 years. The conclusions offer useful guidance for infection control within our institution. However, they may not apply directly to other settings.

Second, the resistance analysis did not cover all drugs used in clinical practice. Notably, carbapenems were not included in the scope of this analysis, as the study focused on three representative antimicrobial classes (cephalosporins, aminoglycosides, and fluoroquinolones). We acknowledge that the exclusion of carbapenem susceptibility data is a limitation, given the clinical importance of carbapenem resistance in these organisms. Carbapenems—particularly imipenem, meropenem, and ertapenem—are among the most clinically relevant agents for empirical and definitive therapy of severe nosocomial infections caused by *Acinetobacter baumannii*, *Klebsiella pneumoniae*, and *Pseudomonas aeruginosa*, and the rapid global expansion of carbapenem-resistant Enterobacterales (CRE) and carbapenem-resistant *A. baumannii* (CRAB) means that any departmental resistance profile that omits carbapenems will inevitably underestimate the true multidrug-resistance burden in critical care and other high-risk settings. The findings reported here should therefore be interpreted as a partial picture, focused on three representative drug classes; we plan to incorporate carbapenem susceptibility data, together with carbapenemase genotyping, in the next phase of our institutional surveillance programme.

Third, the 2020–2024 study period coincided with the COVID-19 pandemic and related public health policies. During this time, healthcare-seeking behavior, hospital case mix, infection control intensity, and antimicrobial prescribing practices all changed substantially. Consequently, the dramatic shifts observed in certain department (just like the *H. influenzae* resurgence in Pediatrics and *P. aeruginosa* decline in PCCM) should be interpreted as pandemic-era phenomena rather than secular AMR trends.

Fourth, we did not perform molecular epidemiological investigations, such as genotyping or resistance gene profiling. As a result, we cannot definitively determine whether the transient amikacin resistance surge in Neurosurgery *K. pneumoniae* represented a clonal outbreak or a shift in aminoglycoside prescribing practices. Future prospective surveillance will incorporate molecular characterization to address this type of question.

Finally, our trend analyses were based on only five annual time points (2020–2024). With only five data points per pathogen–department combination, we have limited ability to detect modest but clinically meaningful trends. Conversely, some statistically significant findings might reflect random year-to-year variation rather than real biological changes. This issue is particularly relevant for strata with small annual isolate counts, where a single unusual year can strongly influence the trend test result. Resistance rate estimates based on fewer than 30 isolates should be interpreted with caution, as reflected by the wide 95% confidence intervals shown in Supplementary Table S2.

Despite these limitations, several signals reached statistical significance. These include the marked oscillation in amikacin resistance among Neurosurgery *K. pneumoniae* (*p* < 0.001) and the progressive changes in *H. influenzae* and *P. aeruginosa* proportions (both *p* < 0.05). This suggests those signals are robust. However, the absence of a statistically significant linear trend for other pathogen–department combinations should not be taken as strong evidence that nothing changed. Detecting smaller, more gradual shifts would require longer surveillance periods with more detailed time resolution (for example, monthly or quarterly data).

## 5 Conclusion

We carried out a five-year surveillance study at our institution. A total of 22,201 clinical isolates from six major departments were included. The results show how pathogen distribution and antimicrobial resistance changed during the COVID-19 pandemic.

Pathogen types varied by department. The ICU carried the highest resistance burden. Importantly, detection trends and resistance trends did not exhibit parallel annual fluctuations. This finding highlights a limitation of using any single metric by itself. Several temporal patterns reached statistical significance. These included the resurgence of *H. influenzae* in Pediatrics, the decline of *P. aeruginosa* in PCCM, and the marked fluctuation in amikacin resistance among Neurosurgery *K. pneumoniae*. However, these patterns were more likely caused by disruptions from the COVID-19 pandemic than by long-term changes in resistance. Longer-term follow-up will be needed to tell temporary fluctuations apart from sustained trends.

The department-specific resistance profiles we documented provide a useful baseline for the post-pandemic period. They can help guide empiric therapy and inform antimicrobial stewardship. Future work should focus on building a stratified management framework. Such a framework should integrate multiple dimensions: epidemiological trends, microbiological data (including resistance phenotypes and genotypes), and clinical outcomes. This would allow for dynamic, evidence-based adjustments to strategy. Sustainable use of antimicrobials depends on two things. One is continuous monitoring of how resistance evolves. The other is timely translation of surveillance data into clinical practice.

## Data Availability

The original contributions presented in the study are included in the article/Supplementary material, further inquiries can be directed to the corresponding author.
