# Supplementary material for: Antimicrobial resistance trends among dominant pathogens in six clinical departments of the Fourth Affiliated Hospital of Guangxi Medical University, 2020–2024
Source: Front Public Health. 2026 Jun 11;14:1836417. doi: 10.3389/fpubh.2026.1836417 (PMC13294266; doi:10.3389/fpubh.2026.1836417)
Supplement: Supplementary file 1 [file Supplementary_file_1.pdf]

Supplementary Document S1: Verification Evidence for CLSI Breakpoint Usage

This document provides verification evidence confirming that the clinical microbiology laboratory at Liuzhou Workers' Hospital used the CLSI M100 S32(not CLSI 2010—during the 2020–2024 study period).

Evidence 1: Published Institutional Surveillance Publication

The following peer-reviewed publication, authored by the clinical microbiology laboratory team of our hospital, covers a subset of the same study period (2020–2022):

Li MW, Wei LH, Luo GL, et al. Surveillance of bacterial resistance in Liuzhou Workers' Hospital, Guangxi from 2020 to 2022 [J]. Chinese Journal of Infection and Chemotherapy, 2025, 25(2): 195–202. (in Chinese)

Figure S1. Abstract of the institutional publication.

The abstract explicitly states: "参照 CLSI M100 32nd 2022 年版药敏折点标准判读结果" (Translation: "Susceptibility results were interpreted according to the CLSI M100 32nd Edition (2022)
breakpoints.")

2020—2022 年广西柳州市工人医院细菌耐药性监测

李梦薇， 韦柳华， 罗国兰， 朱虹贞， 林盛张， 陈丽坤， 蒋利君， 王海霞

摘要： **目的** 了解 2020—2022 年连续 3 年广西柳州市工人医院临床分离菌对常见抗菌药物的耐药性与敏感性。**方法** 使用 VITEK-2 Compact 或纸片扩散法或 E 试验法进行药敏试验，参照 CLSI M100 32nd 2022 年版药敏折点标准判读结果，采用 WHONET 5.6 软件进行分析。**结果** 该院共收集到临床分离株 26 254 株，其中革兰阳性菌 27.9%，革兰阴性菌 72.1%。甲氧西林耐药葡萄球菌 MRSA 和 MRCNS 的检出率分别为 20.0% 和 72.2%。甲氧西林耐药株（MRSA 和 MRCNS）对大多数抗菌药物耐药率高于甲氧西林敏感株（MSSA 和 MSCNS）；未发现有万古霉素、利奈唑胺和替加环素的耐药菌株。屎肠球菌对大多常用抗菌药物耐药率普遍高于粪肠球菌，均未发现两者对万古霉素耐药的菌株，但有少数菌株对利奈唑胺耐药。非脑膜炎肺炎链球菌中儿童株和成人株分别为 691 株和 123 株；两组菌株中 PRSP 分别为 0.4% 和 1.6%，但均未发现有中介株。耐碳青霉烯类肺炎克雷伯菌（CRKpn）的检出率三年间分别为 1.2%、1.2%、13.8%；而铜绿假单胞菌和鲍曼不动杆菌中耐碳青霉烯类菌株（CRPae 和 CRAba）三年间分别为 10.7%、17.5%、14.3% 和 68.4%、75.2%、77.3%。1 269 株流感嗜血杆菌中儿童株和成人株各为 84.6% 和 15.4%，两组菌株中 β 内酰胺酶的检出率分别为 39.4% 和 46.8%；产酶株对氨苄西林耐药，但在不产酶株中已经发现有少数的氨苄西林耐药（BLNAR）株，其检出率为 27.0%。**结论** 该院细菌对抗菌药物的耐药形势严峻，尤其是碳青霉烯类耐药革兰阴性杆菌（CRO）菌株的检出率较高，应加强院感防控措施和临床抗菌药物管理，及 CRO 的主动筛查。临床应多送检疑似感染部位的标本，在药敏报告发出前首先经验用药，待收到药敏报告后，再根据药敏报告合理选用抗菌药物。

关键词： 耐药监测； 药敏试验； 全自动微生物质谱检测仪； 碳青霉烯类耐药菌； 抗菌药物
中图分类号：R378 **文献标识码：**A **文章编号：**1009-7708（2025）02-0195-08
DOI: 10.16718/j.1009-7708.2025.02.012

Surveillance of bacterial resistance in Liuzhou Workers' Hospital, Guangxi from 2020 to 2022

LI Mengwei, WEI Liuhua, LUO Guolan, ZHU Hongzhen, LIN Shengzhang, CHEN Likun, JIANG Lijun, WANG Haixia（*Department of Laboratory Medicine, the Fourth Affiliated Hospital of Guangxi Medical University, Liuzhou Workers' Hospital, Liuzhou Guangxi 545005, China*）

Figure S2. Methods section of the institutional publication.

1　材料与方法

1.1　材料

1.1.1　菌株收集　收集广西柳州市工人医院 2020 年至 2022 年临床分离株（剔除同一患者的重复分离株）共 26 254 株。剔除非无菌体液标本分离的凝固酶阴性葡萄球菌和草绿色链球菌。

1.1.2　培养基和药敏纸片　药敏试验用 MH 培养基、药敏纸片均为英国 OXOID 公司商品，青霉素、万古霉素 E 试验条为温州康泰生物科技有限公司商品，细菌鉴定与药敏试验采用全自动细菌鉴定及药敏分析仪 VITEK-2、全自动微生物质谱检测仪 VITEK MS 为法国生物梅里埃公司商品。

1.2　方法

1.2.1　药敏试验　对常见细菌的药敏试验采用全自动药敏分析仪，对少数仪器法折点不覆盖的抗生素如抗肺炎链球菌的克林霉素和青霉素，采用纸片扩散法或 / 和 E 试验法进行补充，结果阅读参照 CLSI 折点标准[1-2]；替加环素判读参照美国食品药品监督管理局（FDA）折点标准[3]，头孢哌酮-舒巴坦结果判读参照头孢哌酮折点[4]。

1.2.2　质控菌株　金黄色葡萄球 ATCC 25923/29213，大肠埃希菌 ATCC 25922，铜绿假单胞菌

The Methods section documents the institutional practice: "结果阅读参照 CLSI 折点标准" (Translation: "Results were interpreted with reference to CLSI breakpoint standards.")

Summary

The published institutional surveillance report confirms that:

The same clinical microbiology laboratory that generated the data for our study used CLSI M100 32nd Edition (2022)—a current, not outdated, CLSI standard.

The laboratory's standard practice was to reference CLSI breakpoint standards for routine susceptibility reporting.

This practice covers the same study period (2020–2022) as our manuscript (2020–2024).

· 论著 ·

2020—2022 年广西柳州市工人医院细菌耐药性监测

李梦薇，韦柳华，罗国兰，朱虹贞，林盛张，陈丽坤，蒋利君，王海霞

摘要：目的 了解 2020—2022 年连续 3 年广西柳州市工人医院临床分离菌对常见抗菌药物的耐药性与敏感性。**方法** 使用 VITEK-2 Compact 或纸片扩散法或 E 试验法进行药敏试验，参照 CLSI M100 32nd 2022 年版药敏折点标准判读结果，采用 WHONET 5.6 软件进行分析。**结果** 该院共收集到临床分离株 26 254 株，其中革兰阳性菌 27.9%，革兰阴性菌 72.1%。甲氧西林耐药葡萄球菌 MRSA 和 MRCNS 的检出率分别为 20.0% 和 72.2%。甲氧西林耐药株（MRSA 和 MRCNS）对大多数抗菌药物耐药率高于甲氧西林敏感株（MSSA 和 MSCNS）；未发现有万古霉素、利奈唑胺和替加环素的耐药菌株。屎肠球菌对大多常用抗菌药物耐药率普遍高于粪肠球菌，均未发现两者对万古霉素耐药的菌株，但有少数菌株对利奈唑胺耐药。非脑膜炎肺炎链球菌中儿童株和成人株分别为 691 株和 123 株；两组菌株中 PRSP 分别为 0.4% 和 1.6%，但均未发现有中介株。耐碳青霉烯类肺炎克雷伯菌（CRKpn）的检出率三年间分别为 1.2%、1.2%、13.8%；而铜绿假单胞菌和鲍曼不动杆菌中耐碳青霉烯类菌株（CRPae 和 CRAba）三年间分别为 10.7%、17.5%、14.3% 和 68.4%、75.2%、77.3%。1 269 株流感嗜血杆菌中儿童株和成人株各为 84.6% 和 15.4%，两组菌株中 β 内酰胺酶的检出率分别为 39.4% 和 46.8%；产酶株对氨苄西林耐药，但在不产酶株中已经发现有少数的氨苄西林耐药（BLNAR）株，其检出率为 27.0%。**结论** 该院细菌对抗菌药物的耐药形势严峻，尤其是碳青霉烯类耐药革兰阴性杆菌（CRO）菌株的检出率较高，应加强院感防控措施和临床抗菌药物管理，及 CRO 的主动筛查。临床应多送检疑似感染部位的标本，在药敏报告发出前首先经验用药，待收到药敏报告后，再根据药敏报告合理选用抗菌药物。

关键词： 耐药监测；药敏试验；全自动微生物质谱检测仪；碳青霉烯类耐药菌；抗菌药物
中图分类号：R378 **文献标识码：**A **文章编号：**1009-7708（2025）02-0195-08
DOI: 10.16718/j.1009-7708.2025.02.012

Surveillance of bacterial resistance in Liuzhou Workers' Hospital, Guangxi from 2020 to 2022

LI Mengwei, WEI Liuhua, LUO Guolan, ZHU Hongzhen, LIN Shengzhang, CHEN Likun, JIANG Lijun, WANG Haixia（*Department of Laboratory Medicine, the Fourth Affiliated Hospital of Guangxi Medical University, Liuzhou Workers' Hospital, Liuzhou Guangxi* 545005*, China*）

Abstract: Objective To understand the changing profiles of antimicrobial susceptibility of the bacterial strains isolated from patients at Liuzhou Workers' Hospital in Guangxi from 2020 to 2022. **Methods** The bacteria were isolated, identified, and underwent antimicrobial susceptibility testing using VITEK 2 Compact, disk diffusion method, or E-test. The results were interpreted according to the breakpoints recommended by CLSI M100 32nd Edition in 2022. The data were analyzed using WHONET 5.6 software. **Results** A total of 26 254 nonduplicate strains were collected from 2020 to 2022, including Gram-positive bacteria (27.9%) and gram-negative bacteria (72.1%). The prevalence of methicillin-resistant strains was 20.0% in *S. aureus* (MRSA), and 72.2% in coagulase-negative *Staphylococcus* (MRCNS). Methicillin-resistant staphylococcal strains were more resistant to most antimicrobial agents than methicillin-susceptible strains (MSSA and MSCNS). None of the staphylococcal strains was resistant to vancomycin, linezolid or tigecycline. *Enterococcus faecium* strains showed higher resistance rates to most antimicrobial agents than *Enterococcus faecalis*. None of enterococcal strains was resistant to vancomycin. A few enterococcal strains were resistant to linezolid. Overall, 691 strains of the non-meningitis

基金项目：广西壮族自治区卫生健康委员会自筹课题（Z20200152）。
作者单位：广西医科大学第四附属医院柳州市工人医院医学检验科，广西柳州 545005。
第一作者简介：李梦薇（1981—），女，本科，副主任技师，主要从事细菌耐药监测及多重耐药菌耐药机制研究。
通信作者：韦柳华，E-mail：weiliuhua2005@163.com。

中国知网 https://www.cnki.net

Streptococcus pneumoniae were isolated from children and 123 strains were isolated from adults. The prevalence of penicillin-resistant *S. pneumoniae* (PRSP) was 0.4% in the strains from children and 1.6% in the strains from adults. None of *S. pneumoniae* strains was intermediate to penicillin. The prevalence of carbapenem-resistant *Klebsiella pneumoniae* (CRKpn) was 1.2%, 1.2%, and 13.8% in 2020, 2021, and 2022, respectively. The prevalence of carbapenem-resistant *P. aeruginosa* (CRPae) and carbapenem-resistant *Acinetobacter baumannii* (CRAba) was 10.7% and 68.4% in 2020, 17.5% and 75.2% in 2021, 14.3% and 77.3% in 2022, respectively. About 84.6% of the 1 269 strains of *Haemophilus influenzae* were isolated from children and 15.4% isolated from adults. The prevalence of beta-lactamase-producing strains was 39.4% in the isolates from children and 46.8% in the isolates from adults. The β-lactamase-producing *H. influenzae* was resistant to ampicillin. Furthermore, some β-lactamase-nonproducing ampicillin-resistant (BLNAR) *H. influenzae* strains (27.0%) were also identified. **Conclusions** Antimicrobial resistance is still serious in this hospital, especially high prevalence of carbapenem-resistant organisms (CRO). Hospital infection prevention and control measures, antibiotic stewardship, and proactive CRO screening should be strengthened. More clinical specimens should be collected for suspected infections. Antimicrobial treatment should be prescribed empirically in time and adjusted when the results of antimicrobial susceptibility testing are available.

Keywords: antimicrobial resistance surveillance, antimicrobial susceptibility testing, matrix-assisted laser desorption/ionization time-of-flight mass spectrometry, carbapenem-resistant organism, antimicrobial agent

抗菌药物的广泛应用和不合理的滥用，导致细菌耐药已成为全球持续关注的问题之一，特别是多重耐药菌的检出率持续上升给临床重症患者的治疗带来极大困难。细菌耐药监测已成为我们了解细菌耐药情况、指导临床规范用药的重要手段，现将广西柳州市工人医院 2020 年至 2022 年连续 3 年临床分离菌的分布和耐药性监测结果报道如下。

1 材料与方法

1.1 材料

1.1.1 菌株收集 收集广西柳州市工人医院 2020 年至 2022 年临床分离株（剔除同一患者的重复分离株）共 26 254 株。剔除非无菌体液标本分离的凝固酶阴性葡萄球菌和草绿色链球菌。

1.1.2 培养基和药敏纸片 药敏试验用 MH 培养基、药敏纸片均为英国 OXOID 公司商品，青霉素、万古霉素 E 试验条为温州康泰生物科技有限公司商品，细菌鉴定与药敏试验采用全自动细菌鉴定及药敏分析仪 VITEK-2、全自动微生物质谱检测仪 VITEK MS 为法国生物梅里埃公司商品。

1.2 方法

1.2.1 药敏试验 对常见细菌的药敏试验采用全自动药敏分析仪，对少数仪器法折点不覆盖的抗生素如抗肺炎链球菌的克林霉素和青霉素，采用纸片扩散法或 / 和 E 试验法进行补充，结果阅读参照 CLSI 折点标准[1-2]；替加环素判读参照美国食品药品监督管理局（FDA）折点标准[3]，头孢哌酮-舒巴坦结果判读参照头孢哌酮折点[4]。

1.2.2 质控菌株 金黄色葡萄球 ATCC 25923/29213，大肠埃希菌 ATCC 25922，铜绿假单胞菌 ATCC 27853，流感嗜血杆菌 ATCC 49247/9007，粪肠球菌 ATCC 29212，肺炎链球菌 ATCC 49619。

1.2.3 β 内酰胺酶检测 流感嗜血杆菌和卡他莫拉菌 β 内酰胺酶检测采用头孢硝噻吩纸片法检测，结果为红色者为产酶阳性。超广谱 β 内酰胺酶（ESBL）的检测采用 CLSI 推荐的酶抑制剂增强确证试验[1]。使用头孢噻肟、头孢他啶、头孢泊肟及相应的含 β 内酰胺酶抑制剂复方制剂，若其中任何一组含酶抑制剂复方制剂的 MIC 值与其单药的 MIC 值比较，降低≥3 个倍比稀释浓度，即确认产 ESBL。

1.2.4 青霉素不敏感肺炎链球菌检测 所有分离到的肺炎链球菌均采用青霉素 E 试验法检测其最低抑菌浓度（MIC），按 CLSI M100 标准判定为青霉素敏感（PSSP）、中介（PISP）或耐药（PRSP）[1]。

1.2.5 碳青霉烯类耐药革兰阴性杆菌（CRO）的确认 CRO 主要包括碳青霉烯类耐药肠杆菌目细菌（CRE）、碳青霉烯类耐药铜绿假单胞菌（CRPae）和碳青霉烯类耐药鲍曼不动杆菌（CRAba）。CRO 菌株的确认包括对 CRE 菌株的确认。即细菌对包括亚胺培南、美罗培南、厄他培南、多立培南等中的任一种碳青霉烯类抗生素耐药者均可被认为是 CRO 菌株。但 CRO 为除外变形杆菌属、摩根菌属、普鲁威登菌属对亚胺培南耐药者、及除外铜绿假单胞菌和鲍曼不动杆菌对厄他培南耐药者[5]。

1.2.6 数据分析 采用 WHONET 5.6 软件进行数据分析。

2 结果

2.1 细菌分布

2020 年至 2022 年我院共分离出临床菌株

中国知网 https://www.cnki.net

26 254 株，其中革兰阳性菌 7 323 株（27.9%），革兰阴性菌 18 931 株（72.1%）。呼吸道标本（20.6%）为所有标本中最多者。见表 1、表 2。

2.2 革兰阳性球菌药敏试验

2.2.1 葡萄球菌属 3 306 株葡萄球菌中，甲氧西林耐药金黄色葡萄球菌（MRSA）占 20.0%（401/2 008），甲氧西林耐药凝固酶阴性葡萄球菌（MRCNS）占 72.2%（937/1 298）。MRSA 和 MRCNS 对氨基糖苷类、克林霉素、大环内酯类和喹诺酮类抗菌药物的耐药率高于甲氧西林敏感株（MSSA 和 MSCNS）。MRSA 对庆大霉素、左氧氟沙星、甲氧苄啶-磺胺甲噁唑耐药率（3.8%、9.5%、7.3%）显著低于 MRCNS（28.8%、63.4%、32.9%）。MRSA 和 MRCNS 均未发现对万古霉素、利奈唑胺和替加环素耐药的菌株。见表 3。

2.2.2 肠球菌属 1 825 株肠球菌属细菌中粪肠球菌为 54.1%（988/1 825），屎肠球菌为 34.0%（621/1 825），余为其他肠球菌。粪肠球菌对大多数常用抗菌药物的耐药率普遍低于屎肠球菌。前者对青霉素和氨苄西林的耐药率较低，分别为 1.0% 和 0.6%。后者屎肠球菌对青霉素和氨苄西林的耐药率高，分别为 90.0% 和 87.4%。粪肠球菌对高浓度庆大霉素和高浓度链霉素耐药率分别为 38.0% 和 25.4%；屎肠球菌对上述两种高浓度氨基糖苷类抗菌药物的耐药率分别为 71.2% 及 64.1%。粪肠球菌和屎肠球菌对利奈唑胺的耐药率分别为 2.0% 和 0.5%，但两者都未发现万古霉素耐药株。见表 4。

表 1 2020—2022 年主要分离的细菌种类

Table 1 Species distribution of bacterial isolates from 2020 to 2022

Organism	No. of strains	Proportion/%
E. coli	5 766	22.0
Klebsiella spp	3 211	12.2
P. aeruginosa	2 029	7.7
S. aureus	2 008	7.6
Enterococcus spp	1 825	7.0
Acinetobacter spp	1 779	6.8
Coagulase negative *Staphylococcus* [a]	1 298	4.9
Haemophilus influenzae	1 269	4.8
Moraxella catarrhalis	1 030	3.9
Streptococcus pneumoniae	815	3.1
S. maltophilia	720	2.7
Proteus spp	705	2.7
Enterobacter spp	680	2.6
Beta-haemolytic *Streptococcus*（Group B）	618	2.4
S. viridans [a]	490	1.9
Citrobacter spp	300	1.1
Serratia spp	223	0.8
Morganella spp	186	0.7
Other *Pseudomonas* species	166	0.6
Aeromonas spp	163	0.6
Burkholderia spp	94	0.4
Salmonella spp	79	0.3
Providencia spp	44	0.2
Others [b]	756	2.9
Total	26 254	100

[a] Isolates from blood，cerebrospinal fluid，or other sterile body fluids.
[b] Including *Elizabethkingia meningosepticum*，*Chryseobacterium* spp，*Pantoea* spp，*Raoultella* spp，*Alcaligenes* spp，*Aerococcus* spp，*Flavobacterium* spp，*Achromobacter* spp，*Chryseomonas luteola*，*Vibrio* spp，*Bacillus* spp，and other *Streptococcus* species.

表 2 2020—2022 年主要分离菌的变迁和排序

Table 2 Changing profiles of major bacterial species isolated from patients from 2020 to 2022

Rank	2020（*n*=7 071）			2021（*n*=9 194）			2022（*n*=9 989）		
	Bacteria	No. of strains	%	Bacteria	No. of strains	%	Bacteria	No. of strains	%
1	*E.coli*	1 586	22.4	*E. coli*	2 053	22.3	*E. coli*	2 127	21.3
2	*Klebsiella*	858	12.1	*Klebsiella*	1 143	12.4	*Klebsiella*	1 210	12.1
3	*P. aeruginosa*	597	8.4	*P. aeruginosa*	703	7.6	*S. aureus*	822	8.2
4	*S. aureus*	534	7.6	*S. aureus*	652	7.1	*P. aeruginosa*	729	7.3
5	*Enterococcus*	524	7.4	*Enterococcus*	623	6.8	*Enterococcus*	678	6.8
6	*A. baumannii*	433	6.1	*A. baumannii*	614	6.7	*H. influenzae*	643	6.4
7	CNS	416	5.9	CNS	452	4.9	*A. baumannii*	594	5.9
8	*M. catarrhalis*	264	3.7	*H. influenzae*	448	4.9	CNS	430	4.3
9	*Enterobacter*	212	3.0	*M. catarrhalis*	346	3.8	*M. catarrhalis*	420	4.2
10	*S. pneumoniae*	212	3.0	*S. pneumoniae*	260	2.8	*S. pneumoniae*	343	3.4
11	*S. maltophilia*	192	2.7	*S. maltophilia*	289	3.1	*S. maltophilia*	254	2.5
12	*Salmonella*	17	0.2	*Salmonella*	27	0.3	*Salmonella*	35	0.4
	Others	1 226	17.3	Others	1 584	17.2	Others	1 704	17.1
	Total	7 071	100	Total	9 194	100	Total	9 989	100

CNS，coagulase-negative *Staphylococcus*.

中国知网 https://www.cnki.net

表 3　2020—2022 年葡萄球菌对抗菌药物的耐药率和敏感率

Table 3　Susceptibility of *Staphylococcus* strains to antimicrobial agents from 2020 to 2022

（%）

Antimicrobial agent	MRSA（*n*=401）		MSSA（*n*=1 607）		MRCNS（*n*=937）		MSCNS（*n*=361）	
	R	S	R	S	R	S	R	S
Penicillin G	100	0	84.4	15.6	100	0	65.0	35.0
Oxacillin	100	0	0	100	100	0	0	100
Gentamicin	3.8	92.9	2.5	95.3	28.8	57.5	0.3	96.5
Clindamycin	45.6	54.4	11.7	87.9	36.7	62.0	12.3	87.1
Erythromycin	67.5	32.2	26.8	72.3	80.3	19.1	48.1	50.3
Vancomycin	0	100	0	100	0	100	0	100
Linezolid	0	100	0	100	0	100	0	100
Tigecycline	0	100	0	100	0	100	0	100
Rifampin	7.3	72.9	1.3	95.9	15.6	83.5	2.6	96.8
Levofloxacin	9.5	88.7	7.6	92.1	63.4	35.4	6.7	92.7
Trimethoprim-sulfamethoxazole	7.3	92.7	7.2	92.8	32.9	67.1	12.5	87.5

表 4　2020—2022 年粪肠球菌和屎肠球菌对抗菌药物的耐药率和敏感率

Table 4　Susceptibility of *E. faecalis* and *E. faecium* to antimicrobial agents from 2020 to 2022

（%）

Antimicrobial agent	*E. faecalis*（*n*=988）		*E. faecium*（*n*=621）	
	R	S	R	S
Penicillin G	1.0	99.0	90.0	10.0
Ampicillin	0.6	99.4	87.4	12.6
Gentamicin-high	38.0	62.0	71.2	28.8
Streptomycin-high	25.4	74.6	64.1	35.9
Vancomycin	0	100	0	100
Linezolid	2.0	97.0	0.5	99.5
Levofloxacin	24.2	74.0	86.7	8.5
Tetracycline	82.1	17.9	74.3	25.4
Nitrofurantoin	0.5	98.3	36.6	17.1

2.2.3 链球菌属　肺炎链球菌 815 株中，814 株为非脑膜炎株，1 株为脑膜炎株（儿童株）。该株脑膜炎肺炎链球菌用 E 试验方法进行青霉素、美罗培南、头孢曲松和头孢噻肟的药敏结果显示，青霉素 MIC 0.06 mg/L（S），美罗培南 MIC 0.06 mg/L（S），头孢曲松 MIC 0.25 mg/L（S）和头孢噻肟 MIC 0.5 mg/L（S）。814 株非脑膜炎肺炎链球菌株中，儿童株 691 株，成人株 123 株，儿童株 PSSP 和 PRSP 检出率分别为 99.6%、0.4%，成人株 PSSP 和 PRSP 检出率分别为 98.4%、1.6%；两组人群中均没有检出 PISP 株。药敏结果显示儿童株和成人株对红霉素和克林霉素的耐药率高（≥90.4%）。除 1 株成人株对左氧氟沙星耐药外，其余菌株对左氧氟沙星和莫西沙星几乎无耐药株。未发现对万古霉素和利奈唑胺耐药的肺炎链球菌。B 群 β 溶血链球菌 618 株中，未发现青霉素耐药株。分离自血液、脑脊液和其他无菌体液的 490 株草绿色链球菌中，4.1% 的菌株对青霉素耐药。草绿色链球菌对红霉素和克林霉素耐药率较高，均≥57.2%。未发现利奈唑胺和万古霉素耐药链球菌。见表 5、表 6。

2.3　革兰阴性菌药敏试验

2.3.1 肠杆菌目细菌　大肠埃希菌对头孢曲松和头孢呋辛的耐药率较高，分别为 43.1% 和 42.8%，但对头孢他啶和头孢吡肟的耐药率各为 16.8% 和 16.7%。包括克雷伯菌属在内的其他肠杆菌目细菌对头孢曲松的耐药率≤28.3%，对头孢他啶和头孢吡肟的耐药率≤19.0%。肠杆菌目细菌对酶抑制剂复方制剂的耐药率均≤13.5%。除了克雷伯菌属和枸橼酸杆菌属对亚胺培南（5.5% 和 2.7%）和美罗培南（2.1% 和 2.6%）2 种碳青霉烯类抗生素的耐药率相对稍高外，其余肠杆菌目细菌对上述 2 种碳青霉烯类抗生素耐药率仍很低，≤1.8%。除大肠埃希菌对左氧氟沙星的耐药率为 49.1%、变形杆菌属的耐药率为 36.3% 外，肠杆菌目细菌对左氧氟沙星的耐药率≤25.3%。该目细菌对阿米卡星和替加环素耐药率分别≤5.1% 和≤7.7%。79 株沙门菌属细菌，主要是鼠伤寒沙门菌血清型 50 株（63.3%）；肠炎沙门菌血清型 13 株（16.5%）；另 16 株血清型分别为 D 群沙门菌 6.3%（5/79）、布利丹沙门菌 3.8%（3/79）、C2 群沙门菌 3.8%（3/79）、斯坦利沙门菌 2.5%（2/79）、丙型副伤寒沙门菌 1.3%（1/79）、婴儿沙门菌 1.3%（1/79）、C1 群沙门菌 1.3%

中国知网　https://www.cnki.net

表 5 2020—2022 年分离自成人和儿童的非脑膜炎肺炎链球菌对抗菌药物的耐药率和敏感率

Table 5 Susceptibility of non-meningitis *S. pneumoniae* strains isolated from children and adults to antimicrobial agents from 2020 to 2022

（%）

Antimicrobial agent	Isolates from children（*n*=691）				Isolates from adults（*n*=123）			
	PSSP（*n*=688）		PRSP（*n*=3）[a]		PSSP（*n*=121）		PRSP（*n*=2）[a]	
	R	S	R	S	R	S	R	S
Penicillin G	0	100	3	0	0	100	2	0
Vancomycin	0	100	0	3	0	100	0	2
Linezolid	0	100	0	3	0	100	0	2
Erythromycin	99.0	1.0	3	0	92.9	7.1	2	0
Clindamycin	98.0	1.7	3	0	90.4	8.6	2	0
Trimethoprim-sulfamethoxazole	59.8	17.5	2	0	54.3	32.4	2	0
Levofloxacin	0	100	0	3	0	100	1	1
Moxifloxacin	0	100	0	3	0	100	0	2
Chloramphenicol	5.2	94.8	0	3	8.7	91.3	0	2

[a] Number of strains is presented instead of percentage.

表 6 草绿色链球菌和 B 群链球菌对抗菌药物的耐药率和敏感率

Table 6 Susceptibility of *S.viridans* and *S.agalactiae* isolates to antimicrobial agents

（%）

Antimicrobial agent	*S. viridans*（*n*=490）		*S. agalactiae*（*n*=618）	
	R	S	R	S
Penicillin	4.1	90.0	0	100
Erythromycin	59.8	38.2	NA	NA
Clindamycin	57.2	42.4	25.4	74.1
Cefotaxime	4.5	95.3	NA	NA
Vancomycin	0	100	0	100
Linezolid	0	100	0	100
Levofloxacin	17.4	81.8	19.7	79.2
Chloramphenicol	3.6	96.4	NA	NA

NA，not available.

(1/79)。主要 2 种沙门菌属细菌对头孢曲松、甲氧苄啶-磺胺甲噁唑耐药率≥38.5%，对亚胺培南和美罗培南高度敏感。见表 7、表 8。

2.3.2 不发酵糖革兰阴性菌 铜绿假单胞菌除对头孢哌酮-舒巴坦、哌拉西林-他唑巴坦和环丙沙星的耐药林为 12.4%、10.3% 和 10.9% 外，对包括亚胺培南和美罗培南在内的所有抗菌药物的耐药率均≤8.5%。鲍曼不动杆菌除对米诺环素、替加环素、黏菌素耐药率分别为 13.6%、1.1% 和 0.4% 外，对包括亚胺培南和美罗培南在内的其他受试抗菌药物的耐药率高，耐药率大多≥54.1%；其中对亚胺培南和美罗培南的耐药率分别高达 71.3% 和 71.7%；对头孢哌酮-舒巴坦和哌拉西林-他唑巴

表 7 2020—2022 年肠杆菌目细菌对抗菌药物的耐药率和敏感率

Table 7 Susceptibility of *Enterobacterales* to antimicrobial agents from 2020 to 2022

（%）

Antimicrobial agent	*E. coli*（*n*=5 766）		*Klebsiella*（*n*=3 211）		*Proteus*（*n*=705）		*Enterobacter*（*n*=680）		*Citrobacter*（*n*=300）		*Serratia*（*n*=223）		*Morganella*（*n*=186）	
	R	S	R	S	R	S	R	S	R	S	R	S	R	S
Amikacin	1.5	98.4	5.1	94.8	0.7	99.3	0.3	99.1	0.7	99.0	0	100	1.1	98.9
Imipenem	1.1	98.8	5.5	94.5	0	100	1.8	98.2	2.7	97.3	0.9	99.1	0	100
Meropenem	0.5	99.5	2.1	97.9	0	100	1.5	96.9	2.6	97.4	0.6	99.4	0.9	99.1
Cefepime	16.7	73.9	15.1	83.3	4.7	90.0	4.2	93.0	4.0	94.0	1.8	96.9	1.5	95.6
Ceftazidime	16.8	73.9	19.0	77.9	2.6	96.4	17.8	81.0	15.0	83.3	3.1	95.6	8.6	87.1
Ceftriaxone	43.1	56.7	28.3	71.5	26.3	72.0	23.3	76.4	17.3	82.7	9.2	90.3	11.8	82.3
Cefuroxime	42.8	52.1	32.9	64.5	46.4	52.4	39.5	38.1	32.0	62.7	92.1	1.6	86.3	5.4
Cefoperazone-sulbactam	4.1	93.3	10.5	86.8	0.8	98.0	13.5	83.9	6.8	90.9	5.7	92.4	7.3	90.5
Cefoxitin	10.0	84.2	22.4	75.6	3.3	90.5	95.0	4.3	36.9	54.3	13.4	35.6	17.8	19.2
Piperacillin-tazobactam	3.8	93.2	11.9	83.3	0.1	98.1	10.8	84.4	6.5	86.1	0	98.7	2.2	97.3
Levofloxacin	49.1	18.3	22.2	57.0	36.3	51.4	8.5	78.3	11.0	77.0	4.9	91.1	25.3	61.3
Tigecycline	0	100	0.2	99.7	7.7	34.6	0.2	99.8	0	100	0	100	0.9	92.0
Trimethoprim-sulfamethoxazole	52.8	47.2	27.1	72.9	61.4	38.6	14.1	85.9	10.7	89.3	1.3	98.7	38.9	61.1

中国知网 https://www.cnki.net

表 8　2020—2022 年主要沙门菌属对抗菌药物的耐药率和敏感率

Table 8　Susceptibility of major *Salmonella* species to antimicrobial agents from 2020 to 2022

（%）

Antimicrobial agent	*S. typhimurium*（*n*=50）		*S. enteritidis*（*n*=13）	
	R	S	R	S
Ceftriaxone	56.0	44.0	46.2	53.8
Levofloxacin	20.0	32.0	15.4	38.4
Trimethoprim-sulfamethoxazole	42.0	58.0	38.5	61.5
Imipenem	0	100	0	100
Meropenem	0	100	0	100

坦的耐药率为 60.1% 和 74.1%。嗜麦芽窄食单胞菌对左氧氟沙星、甲氧苄啶-磺胺甲噁唑、米诺环素耐药率分别为 6.8%、2.4% 和 0.8%。见表 9。

2.3.3　CRO　CRO 的检出率见表 10。其中碳青霉烯类耐药大肠埃希菌和肺炎克雷伯菌（CREco 和 CRKpn）、碳青霉烯类耐药鲍曼不动杆菌和铜绿假单胞菌（CRAba 和 CRPae）有持续上升的趋势。

2.3.4　流感嗜血杆菌和卡他莫拉菌　流感嗜血杆菌 1 269 株中儿童株 84.6%（1 074/1 269），成人株 15.4%（195/1 269）。β 内酰胺酶的检出率 40.7%，其中儿童株和成人株产酶株检出率各为 39.4% 和 46.8%。β 内酰胺酶阴性氨苄西林耐药（BLNAR）株检出率 27.0%。流感嗜血杆菌产酶株对抗菌药物阿莫西林-克拉维酸、头孢噻肟、氯霉素、四环素和氧氟沙星高度敏感，耐药率≤9.3%，而其中产酶株对氯霉素和四环素耐药率高于不产酶株，儿童株对阿莫西林-克拉维酸耐药率高于成人株。1 030 株卡他莫拉菌中 99.2% 为产酶株，对阿莫西林-克拉维酸、头孢克洛、氯霉素和四环素耐药率较低，分别为 0.2%、2.6%、0.5% 和 2.9%。见表 11。

表 9　2020—2022 年不发酵糖革兰阴性杆菌对抗菌药物的耐药率和敏感率

Table 9　Susceptibility of non-fermentative gram-negative bacilli to antimicrobial agents from 2020 to 2022

（%）

Antimicrobial agent	*P. aeruginosa*（*n*=2 029）		*A. baumannii*（*n*=1 641）		*S. maltophilia*（*n*=735）	
	R	S	R	S	R	S
Amikacin	1.4	97.7	NA	NA	NA	NA
Tobramycin	3.7	95.1	69.3	30.6	NA	NA
Imipenem	8.4	91.0	71.3	28.6	NA	NA
Meropenem	6.7	91.5	71.7	28.3	NA	NA
Cefepime	3.8	90.3	67.3	27.9	NA	NA
Ceftazidime	8.5	87.1	71.8	26.4	NA	NA
Cefoperazone-sulbactam	12.4	76.9	60.1	28.6	NA	NA
Piperacillin-tazobactam	10.3	80.0	74.1	25.6	NA	NA
Ciprofloxacin	10.9	82.4	73.2	26.5	NA	NA
Levofloxacin	NA	NA	68.6	27.5	6.8	91.0
Trimethoprim-sulfamethoxazole	NA	NA	54.1	45.9	2.4	97.0
Minocycline	NA	NA	13.6	48.2	0.8	97.7
Tigecycline	NA	NA	1.1	91.3	NA	NA
Colistin	1.9	0	0.4	98.2	NA	NA

NA，not available.

表 10　2020—2022 年碳青霉烯类耐药革兰阴性杆菌检出率

Table 10　Prevalence of carbapenem-resistant organisms from 2020 to 2022

Organism	CRO isolates in 2020		CRO isolates in 2021		CRO isolates in 2022		Total CRO in 2020-2022	
	Number	%	Number	%	Number	%	Number	%
E. coli	9	0.6	20	1.0	38	1.8	67	1.2
Klebsiella pneumoniae	9	1.2	12	1.2	147	13.8	168	6.0
Citrobacter freundii	2	6.9	3	9.1	2	4.3	7	6.5
Enterobacter cloacae	1	0.5	8	4.0	2	0.9	11	1.8
Pseudomonas aeruginosa	64	10.7	123	17.5	104	14.3	291	14.3
Acinetobacter baumannii	296	68.4	462	75.2	459	77.3	1 217	74.2

CRO，carbapenem-resistant organism.

中国知网　https://www.cnki.net

表 11 2020—2022 年流感嗜血杆菌和卡他莫拉菌对抗菌药物的耐药率和敏感率

Table 11 Susceptibility of *H. influenzae* and *M. catarrhalis* to antimicrobial agents from 2020 to 2022

（%）

Antimicrobial agent	*H. influenzae*（*n*=1 269）								*M. catarrhalis*（*n*=1 030）	
	β-lactamase（+）（*n*=516）		β-lactamase（-）（*n*=753）		Isolates from children（*n*=1 074）		Isolates from adults（*n*=195）		Total（*n*=1 030）	
	R	S	R	S	R	S	R	S	R	S
Ampicillin	97.5	2.5	27.0	72.7	55.0	44.8	58.4	41.6	NA	NA
Amoxicillin-clavulanic acid	5.0	95.0	7.8	92.2	7.3	92.7	3.7	96.3	0.2	99.8
Cefuroxime	32.6	62.9	19.8	76.7	25.0	71.2	24.2	72.1	NA	NA
Cefaclor	39.9	49.6	22.3	73.8	29.3	64.1	28.9	65.8	2.6	93.9
Cefotaxime	0	100	0	100	0	100	0	100	0	100
Chloramphenicol	9.3	89.9	0.1	99.8	3.6	95.9	5.3	94.7	0.5	99.2
Tetracycline	7.9	89.8	2.1	97.4	4.3	94.5	5.8	92.6	2.9	97.0
Ofloxacin	0	100	0	100	0	100	0	100	0	100
Trimethoprim-sulfamethoxazole	70.5	29.1	53.7	46.3	60.9	38.9	58.4	41.6	32.3	66.9

NA，not available.

3 讨论

本组资料显示，26 254 株临床分离菌中革兰阴性菌和革兰阳性菌分别占 72.1% 和 27.9%，与全国细菌耐药监测网报告的数据一致[6-8]。其中大肠埃希菌、克雷伯菌属、铜绿假单胞菌、金黄色葡萄球菌和肠球菌属最为多见。

肺炎链球菌是社区获得性肺炎的常见病原菌，我院 3 年监测非脑膜炎肺炎链球菌 814 株，主要分离自儿科患者，其中 71% 分离自下呼吸道标本，且集中在当年的冬季与次年的春季，推测可能与我院地处南方，广西的温湿度和呼吸道病毒季节性波动造成的呼吸道黏膜损伤有助于肺炎链球菌进入呼吸道、血液系统进而形成侵袭性感染有关[9]。该菌对青霉素仍十分敏感，我院的 PRSP 检出率低，儿童株 0.4%，成人株 1.6%，且无中介株（PISP）。提示青霉素仍可以作为呼吸道肺炎链球菌感染的首选用药之一。但该菌对克林霉素和红霉素的耐药率高，此与全国 CARSS 网 2020 年、2021 年报道的数据[7-8]、以及国内同期报道的数据[10-11]相似。

流感嗜血杆菌最常定植于人体的鼻咽部，黏附于呼吸道上皮细胞，其具有迁移到宿主别的生态位的能力[12]。有研究显示高携带率是流感嗜血杆菌呼吸道感染的前提[13-14]，儿童特别是 0～6 岁的患儿免疫系统尚未发育完善，使得该菌特别容易在原定植部位占据主导或者迁移到其他部位从而导致相关部位的侵袭性感染。本组资料中我院监测到的 1 296 株流感嗜血杆菌主要分离自儿童的呼吸道标本和耳分泌液，很好地印证了这一点。有呼吸道症状的患儿更要关注该菌感染的可能，特别是春夏、秋冬换季的时候。我院 3 年流感嗜血杆菌 β 内酰胺酶检出率历年约在 40% 左右，低于国内同期同类报告[7-8, 14]，但是我院 BLNAR 株的检出率 27%，高于山东省监测网关于 BLNAR 的检出率平均 8.1% 的报道。我院 BLNAR 株高检出率有待进一步研究。

CRO 的出现是全球感染性疾病药物开支负担急剧增加的原因。近年我院包括 CRE 菌株在内的 CRO 菌株如 CRPae、CRAba 有上升的趋势。此外，我院 CRKpn 增长的态势也比较严峻，3 年来肺炎克雷伯菌 CRKpn 的检出率分别为 1.2%、1.2%、13.8%，有明显的上升趋势。此推测可能与近几年来为应对产 ESBL 和产 AmpC 酶耐药菌，我院临床曾大量使用碳青霉烯类药物有关。

参考文献

[1] Clinical and Laboratory Standards Institute. Performance standards for antimicrobial susceptibility testing[S]. M100-S30. Wayne，PA：CLSI，2020.

[2] Clinical and Laboratory Standards Institute. Methods for antimicrobial dilution and disk susceptibility testing of infrequently isolated or fastidious bacteria[S]. M45-S3. Wayne，PA：CLSI，2016.

[3] U.S.Food and Drug Administration. FDA-identified interpretive criteria[EB/OL].[2023-02-08].https：//www.fda.gov/drugs/development-res-ources/tigecycline-injection-products.

[4] JONES R N，BARRY A L，PACKER R R，等 . 关于头孢哌酮-舒巴坦联用的体外抗菌谱、协同作用和对稀释敏感性测试浓度建议的研究 [J]. 临床微生物学杂志，1987，25（9）：1725-1729.

中国知网 https://www.cnki.net

[5] 喻华，徐雪松，李敏，等 . 肠杆菌目细菌碳青霉烯酶的实验室检测和临床报告规范专家共识（第二版）[J]. 中国感染与化疗杂志，2022，22（4）：463-474.
[6] 全国细菌耐药监测网 . 全国细菌耐药监测网 2014—2019 年细菌耐药性监测报告 [J]. 中国感染控制杂志，2021，20（1）：15-31.
[7] 胡付品，郭燕，朱德妹，等 . 2020 年 CHINET 中国细菌耐药监测 [J]. 中国感染与化疗杂志，2021，21（4）：377-387.
[8] 胡付品，郭燕，朱德妹，等 . 2021 年 CHINET 中国细菌耐药监测 [J]. 中国感染与化疗杂志，2022，22（5）：521-530.
[9] 张慧芬，李基明，吴佳音，等 . 儿童侵袭性肺炎链球菌病 35 例临床特征和分离株耐药性分析 [J]. 中国感染与化疗杂志，2021，21（2）：152-157.
[10] 黎昆，王芬，王洁，等 . 2018—2020 年四川遂宁市中心医院细菌耐药性监测 [J]. 中国感染与化疗杂志，2022，22（1）：78-84.
[11] 孟青，崔晓燕，周林涛，等 . 2018—2020 年深圳市儿童医院临床分离细菌分布及耐药性监测 [J]. 中国感染与化疗杂志，2022，22（3）：314-321.
[12] TIKHOMIROVA A，KIDD S P. *Haemophilus influenzae* and *Streptococcus pneumoniae*：living together in a biofilm[J]. Pathog Dis，2013，69（2）：114-126.
[13] ZHU H，WANG A，TONG J，et al. Nasopharyngeal carriage and antimicrobial susceptibility of *Haemophilus influenzae* among children younger than 5 years of age in Beijing，China[J]. BMC Microbiol，2015，15：6.
[14] 孙彦蒙，王梦园，李政，等 . 2017—2019 年山东省临床分离流感嗜血杆菌的耐药性监测 [J]. 中国感染与化疗杂志，2023，23（1）：73-79.

收稿日期：2023-09-07　**修回日期**：2024-09-28

读者·作者·编者

《中国感染与化疗杂志》投稿注意事项

针对本刊作者投稿过程中的常见问题，编辑部特汇总并作如下提示：

1. 正式投稿前，请认真阅读《中国感染与化疗杂志》投稿须知（http://kghl.cbpt.cnki.net/EditorDN/PromptPageInfo.aspx?t=v&c=1），并按照相应格式要求书写。

2. 本刊全部采用线上投稿。请至本杂志官网（http://www.cjic.com.cn/）点击“在线投稿系统”或直接登陆本刊投稿系统网页（http://kghl.cbpt.cnki.net/EditorDN/Quit.aspx）进行投稿。

3. 打开网页后，请在“作者工作区”按提示操作，注册时务必准确填写第一作者及通信作者的详细地址和联系方式（手机号码、E-mail 等）以便后续联系，注册成功后即可上传稿件。

4. 在投稿时，请认真上传单位推荐信（须加盖单位公章）和承诺与确认函（打勾并签名），将 2 份文件以 pdf 或者 jpg 格式在系统中上传，并请保存好原件以供正式录用时提交。经过初审遴选后，编辑部会通过邮件给予作者答复，发送收稿或退稿通知。

5. 当作者收到“《中国感染与化疗杂志》收稿通知”邮件后，请尽快按照收稿通知要求进行相应操作，编辑部收到所有材料后方可开始后续的审稿退修工作。

6. 单位推荐信、承诺与确认函在收稿通知中已有相应模板可供下载。若无法正常下载，还可至“投稿须知”中下载相应模板。

7. 请认真填写承诺与确认函中所有选项，并由第一作者和通信作者签字确认。

8. 单位推荐信请写出所有作者的所在单位，作者的人数和顺序要与文章一致，并加盖单位公章。

9. 为方便编辑，文稿请采用 Word 文档，并在文中标明中英文的文题、摘要（仅论著）、关键词、基金项目（项目编号）、作者单位（具体到科室，包括邮编、城市名）、第一作者简介及通信作者。

10. 编辑部根据审稿意见和编辑规范对来稿进行修改和（或）提出修改意见后退作者修改，若作者 3 个月内不修回，作自动撤稿处理。

中国知网 https://www.cnki.net
