# Supplementary material for: Antimicrobial resistance trends among dominant pathogens in six clinical departments of the Fourth Affiliated Hospital of Guangxi Medical University, 2020–2024
Source: Front Public Health. 2026 Jun 11;14:1836417. doi: 10.3389/fpubh.2026.1836417 (PMC13294266; doi:10.3389/fpubh.2026.1836417)
Supplement: Supplementary file 2 [file Supplementary_file_2.pdf]

Supplementary Table S2. Antimicrobial resistance rates with 95% confidence intervals for predominant pathogens (2020-2024).

Dept	Pathogen	Drug	Year	n	k	%R	95% CI
ICU	A. baumannii	CAZ	2020	111	90	81.1	72.5-87.9
ICU	A. baumannii	CAZ	2021	206	196	95.1	91.2-97.6
ICU	A. baumannii	CAZ	2022	270	259	95.9	92.8-97.9
ICU	A. baumannii	CAZ	2023	285	265	93	89.3-95.7
ICU	A. baumannii	CAZ	2024	234	226	96.6	93.4-98.4
ICU	A. baumannii	TOB	2020	111	88	79.3	70.5-86.4
ICU	A. baumannii	TOB	2021	206	195	94.7	90.6-97.3
ICU	A. baumannii	TOB	2022	270	246	91.1	87.1-94.2
ICU	A. baumannii	TOB	2023	285	256	89.8	85.7-93.1
ICU	A. baumannii	TOB	2024	234	207	88.5	83.7-92.3
ICU	A. baumannii	LEV	2020	111	80	72.1	62.7-80.2
ICU	A. baumannii	LEV	2021	206	187	90.8	86.0-94.4
ICU	A. baumannii	LEV	2022	270	250	92.6	88.8-95.4
ICU	A. baumannii	LEV	2023	285	259	90.9	86.9-94.0
ICU	A. baumannii	LEV	2024	234	214	91.5	87.1-94.7
PCCM	P. aeruginosa	CAZ	2020	165	15	9.1	5.2-14.6
PCCM	P. aeruginosa	CAZ	2021	160	22	13.8	8.8-20.1
PCCM	P. aeruginosa	CAZ	2022	107	9	8.4	4.1-15.0
PCCM	P. aeruginosa	CAZ	2023	99	7	7.1	3.0-13.8
PCCM	P. aeruginosa	CAZ	2024	112	22	19.6	12.7-28.2
PCCM	P. aeruginosa	TOB	2020	165	5	3	1.1-6.7
PCCM	P. aeruginosa	TOB	2021	160	24	15	9.8-21.5
PCCM	P. aeruginosa	TOB	2022	107	3	2.8	0.7-7.6

PCCM	P. aeruginosa	TOB	2023	99	4	4	1.2-9.6
PCCM	P. aeruginosa	TOB	2024	112	2	1.8	0.3-5.9
PCCM	P. aeruginosa	LEV	2020	165	17	10.3	6.1-16.0
PCCM	P. aeruginosa	LEV	2021	160	63	39.4	31.8-47.4
PCCM	P. aeruginosa	LEV	2022	107	27	25.2	17.4-34.5
PCCM	P. aeruginosa	LEV	2023	99	27	27.3	18.8-37.1
PCCM	P. aeruginosa	LEV	2024	112	26	23.2	15.8-32.1
Heme	E. coli	CAZ	2020	27	5	18.5	7.0-36.4
Heme	E. coli	CAZ	2021	38	6	15.8	6.2-30.8
Heme	E. coli	CAZ	2022	33	4	12.1	3.7-27.3
Heme	E. coli	CAZ	2023	48	13	27.1	15.4-41.5
Heme	E. coli	CAZ	2024	51	11	21.6	11.5-35.1
Heme	E. coli	AMK	2020	27	1	3.7	0.2-17.1
Heme	E. coli	AMK	2021	38	1	2.6	0.1-12.5
Heme	E. coli	AMK	2022	33	0	0	0.0-10.3
Heme	E. coli	AMK	2023	48	2	4.2	0.6-13.4
Heme	E. coli	AMK	2024	51	3	5.9	1.4-15.5
Heme	E. coli	LEV	2020	27	15	55.6	36.0-74.0
Heme	E. coli	LEV	2021	38	23	60.5	43.7-75.7
Heme	E. coli	LEV	2022	33	19	57.6	39.5-74.2
Heme	E. coli	LEV	2023	48	23	47.9	33.5-62.6
Heme	E. coli	LEV	2024	51	22	43.1	29.6-57.5
Peds	H. influenzae	CTX	2020	121	0	0	0.0-3.0
Peds	H. influenzae	CTX	2021	323	0	0	0.0-1.2
Peds	H. influenzae	CTX	2022	340	0	0	0.0-1.1
Peds	H. influenzae	CTX	2023	168	0	0	0.0-2.2

Peds	H. influenzae	CTX	2024	217	0	0	0.0-1.7
Peds	H. influenzae	OFX	2020	121	0	0	0.0-3.0
Peds	H. influenzae	OFX	2021	323	0	0	0.0-1.2
Peds	H. influenzae	OFX	2022	340	0	0	0.0-1.1
Peds	H. influenzae	OFX	2023	168	0	0	0.0-2.2
Peds	H. influenzae	OFX	2024	217	0	0	0.0-1.7
Ortho	E. coli	CAZ	2020	85	13	15.3	8.4-24.8
Ortho	E. coli	CAZ	2021	212	43	20.3	15.1-26.3
Ortho	E. coli	CAZ	2022	189	29	15.3	10.5-21.3
Ortho	E. coli	CAZ	2023	218	48	22	16.7-28.1
Ortho	E. coli	CAZ	2024	252	43	17.1	12.6-22.3
Ortho	E. coli	AMK	2020	85	3	3.5	0.9-9.5
Ortho	E. coli	AMK	2021	212	4	1.9	0.6-4.6
Ortho	E. coli	AMK	2022	189	1	0.5	0.0-2.9
Ortho	E. coli	AMK	2023	218	6	2.8	1.1-5.8
Ortho	E. coli	AMK	2024	252	5	2	0.7-4.5

Caption for Supplementary Table S2: *A. baumannii*= Acinetobacter baumannii; *P. aeruginosa*= Pseudomonas aeruginosa; *E. coli*= Escherichia coli; *H. influenzae*= Haemophilus influenzae; *K. pneumoniae*=Klebsiella pneumoniae.

CAZ=Ceftazidime; TOB=Tobramycin; LEV= Levofloxacin; AMK= Amikacin; CTX= Cefotaxime; OFX= Ofloxacin. A. baumannii= Acinetobacter baumannii; P. aeruginosa= Pseudomonas aeruginosa; E. coli= Escherichia coli; H. influenzae= Haemophilus influenzae; K. pneumoniae= K. pneumoniae .
